# Determinants of recovery from post-COVID-19 dyspnoea: analysis of UK prospective cohorts of hospitalised COVID-19 patients and community-based controls

**DOI:** 10.1016/j.lanepe.2023.100635

**Published:** 2023-04-28

**Authors:** Bang Zheng, Giulia Vivaldi, Luke Daines, Olivia C. Leavy, Matthew Richardson, Omer Elneima, Hamish J.C. McAuley, Aarti Shikotra, Amisha Singapuri, Marco Sereno, Ruth M. Saunders, Victoria C. Harris, Linzy Houchen-Wolloff, Neil J. Greening, Paul E. Pfeffer, John R. Hurst, Jeremy S. Brown, Manu Shankar-Hari, Carlos Echevarria, Anthony De Soyza, Ewen M. Harrison, Annemarie B. Docherty, Nazir Lone, Jennifer K. Quint, James D. Chalmers, Ling-Pei Ho, Alex Horsley, Michael Marks, Krishna Poinasamy, Betty Raman, Liam G. Heaney, Louise V. Wain, Rachael A. Evans, Christopher E. Brightling, Adrian Martineau, Aziz Sheikh, PHOSP-COVID Study Collaborative Groupai, K. Abel, H. Adamali, D. Adeloye, O. Adeyemi, R. Adrego, L.A. Aguilar Jimenez, S. Ahmad, N. Ahmad Haider, R. Ahmed, N. Ahwireng, M. Ainsworth, B. Al-Sheklly, A. Alamoudi, M. Ali, M. Aljaroof, A.M. All, L. Allan, R.J. Allen, L. Allerton, L. Allsop, P. Almeida, D. Altmann, M. Alvarez Corral, S. Amoils, D. Anderson, C. Antoniades, G. Arbane, A. Arias, C. Armour, L. Armstrong, N. Armstrong, D. Arnold, H. Arnold, A. Ashish, A. Ashworth, M. Ashworth, S. Aslani, H. Assefa-Kebede, C. Atkin, P. Atkin, R. Aul, H. Aung, L. Austin, C. Avram, A. Ayoub, M. Babores, R. Baggott, J. Bagshaw, D. Baguley, L. Bailey, J.K. Baillie, S. Bain, M. Bakali, M. Bakau, E. Baldry, D. Baldwin, M. Baldwin, C. Ballard, A. Banerjee, B. Bang, R.E. Barker, L. Barman, S. Barratt, F. Barrett, D. Basire, N. Basu, M. Bates, A. Bates, R. Batterham, H. Baxendale, H. Bayes, M. Beadsworth, P. Beckett, M. Beggs, M. Begum, P. Beirne, D. Bell, R. Bell, K. Bennett, E. Beranova, A. Bermperi, A. Berridge, C. Berry, S. Betts, E. Bevan, K. Bhui, M. Bingham, K. Birchall, L. Bishop, K. Bisnauthsing, J. Blaikely, A. Bloss, A. Bolger, C.E. Bolton, J. Bonnington, A. Botkai, C. Bourne, M. Bourne, K. Bramham, L. Brear, G. Breen, J. Breeze, A. Briggs, E. Bright, C.E. Brightling, S. Brill, K. Brindle, L. Broad, A. Broadley, C. Brookes, M. Broome, A. Brown, J. Brown, J.S. Brown, M. Brown, V. Brown, T. Brugha, N. Brunskill, M. Buch, P. Buckley, A. Bularga, E. Bullmore, L. Burden, T. Burdett, D. Burn, G. Burns, A. Burns, J. Busby, R. Butcher, A. Butt, S. Byrne, P. Cairns, P.C. Calder, E. Calvelo, H. Carborn, B. Card, C. Carr, L. Carr, G. Carson, P. Carter, A. Casey, M. Cassar, J. Cavanagh, M. Chablani, T. Chalder, J.D. Chalmers, R.C. Chambers, F. Chan, K.M. Channon, K. Chapman, A. Charalambou, N. Chaudhuri, A. Checkley, J. Chen, Y. Cheng, L. Chetham, C. Childs, E.R. Chilvers, H. Chinoy, A. Chiribiri, K. Chong-James, G. Choudhury, N. Choudhury, P. Chowienczyk, C. Christie, M. Chrystal, D. Clark, C. Clark, J. Clarke, S. Clohisey, G. Coakley, Z. Coburn, S. Coetzee, J. Cole, C. Coleman, F. Conneh, D. Connell, B. Connolly, L. Connor, A. Cook, B. Cooper, J. Cooper, S. Cooper, D. Copeland, T. Cosier, M. Coulding, C. Coupland, E. Cox, T. Craig, P. Crisp, D. Cristiano, M.G. Crooks, A. Cross, I. Cruz, P. Cullinan, D. Cuthbertson, L. Daines, M. Dalton, P. Daly, A. Daniels, P. Dark, J. Dasgin, A. David, C. David, E. Davies, F. Davies, G. Davies, G.A. Davies, K. Davies, M.J. Davies, J. Dawson, E. Daynes, A. De Soyza, B. Deakin, A. Deans, C. Deas, J. Deery, S. Defres, A. Dell, K. Dempsey, E. Denneny, J. Dennis, A. Dewar, R. Dharmagunawardena, N. Diar-Bakerly, C. Dickens, A. Dipper, S. Diver, S.N. Diwanji, M. Dixon, R. Djukanovic, H. Dobson, S.L. Dobson, A.B. Docherty, A. Donaldson, T. Dong, N. Dormand, A. Dougherty, R. Dowling, S. Drain, K. Draxlbauer, K. Drury, H.J.C. drury, P. Dulawan, A. Dunleavy, S. Dunn, C. Dupont, J. Earley, N. Easom, C. Echevarria, S. Edwards, C. Edwardson, H. El-Taweel, A. Elliott, K. Elliott, Y. Ellis, A. Elmer, O. Elneima, D. Evans, H. Evans, J. Evans, R. Evans, R.A. Evans, R.I. Evans, T. Evans, C. Evenden, L. Evison, L. Fabbri, S. Fairbairn, A. Fairman, K. Fallon, D. Faluyi, C. Favager, T. Fayzan, J. Featherstone, T. Felton, J. Finch, S. Finney, J. Finnigan, L. Finnigan, H. Fisher, S. Fletcher, R. Flockton, M. Flynn, H. Foot, D. Foote, A. Ford, D. Forton, E. Fraile, C. Francis, R. Francis, S. Francis, A. Frankel, E. Fraser, R. Free, N. French, X. Fu, J. Fuld, J. Furniss, L. Garner, N. Gautam, J.R. Geddes, J. George, P. George, M. Gibbons, M. Gill, L. Gilmour, F. Gleeson, J. Glossop, S. Glover, N. Goodman, C. Goodwin, B. Gooptu, H. Gordon, T. Gorsuch, M. Greatorex, P.L. Greenhaff, W. Greenhalf, A. Greenhalgh, N.J. Greening, J. Greenwood, H. Gregory, R. Gregory, D. Grieve, D. Griffin, L. Griffiths, A.-M. Guerdette, B. Guillen Guio, M. Gummadi, A. Gupta, S. Gurram, E. Guthrie, Z. Guy, H.H. Henson, K. Hadley, A. Haggar, K. Hainey, B. Hairsine, P. Haldar, I. Hall, L. Hall, M. Halling-Brown, R. Hamil, A. Hancock, K. Hancock, N.A. Hanley, S. Haq, H.E. Hardwick, E. Hardy, T. Hardy, B. Hargadon, K. Harrington, E. Harris, V.C. Harris, E.M. Harrison, P. Harrison, N. Hart, A. Harvey, M. Harvey, M. Harvie, L. Haslam, M. Havinden-Williams, J. Hawkes, N. Hawkings, J. Haworth, A. Hayday, M. Haynes, J. Hazeldine, T. Hazelton, L.G. Heaney, C. Heeley, J.L. Heeney, M. Heightman, S. Heller, M. Henderson, L. Hesselden, M. Hewitt, V. Highett, T. Hillman, T. Hiwot, L.P. Ho, A. Hoare, M. Hoare, J. Hockridge, P. Hogarth, A. Holbourn, S. Holden, L. Holdsworth, D. Holgate, M. Holland, L. Holloway, K. Holmes, M. Holmes, B. Holroyd-Hind, L. Holt, A. Hormis, A. Horsley, A. Hosseini, M. Hotopf, L. Houchen-Wolloff, K. Howard, L.S. Howard, A. Howell, E. Hufton, A.D. Hughes, J. Hughes, R. Hughes, A. Humphries, N. Huneke, E. Hurditch, J. Hurst, M. Husain, T. Hussell, J. Hutchinson, W. Ibrahim, F. Ilyas, J. Ingham, L. Ingram, D. Ionita, K. Isaacs, K. Ismail, T. Jackson, J. Jacob, W.Y. James, W. Jang, C. Jarman, I. Jarrold, H. Jarvis, R. Jastrub, B. Jayaraman, R.G. Jenkins, P. Jezzard, K. Jiwa, C. Johnson, S. Johnson, D. Johnston, C.J. Jolley, D. Jones, G. Jones, H. Jones, I. Jones, L. Jones, M.G. Jones, S. Jones, S. Jose, T. Kabir, G. Kaltsakas, V. Kamwa, N. Kanellakis, s. Kaprowska, Z. Kausar, N. Keenan, S. Kelly, G. Kemp, S. Kerr, H. Kerslake, A.L. Key, F. Khan, K. Khunti, S. Kilroy, B. King, C. King, L. Kingham, J. Kirk, P. Kitterick, P. Klenerman, L. Knibbs, S. Knight, A. Knighton, O. Kon, S. Kon, S.S. Kon, S. Koprowska, A. Korszun, I. Koychev, C. Kurasz, P. Kurupati, C. Laing, H. Lamlum, G. Landers, C. Langenberg, D. Lasserson, L. Lavelle-Langham, A. Lawrie, C. Lawson, A. Layton, A. Lea, O.C. Leavy, D. Lee, J.-H. Lee, E. Lee, K. Leitch, R. Lenagh, D. Lewis, J. Lewis, K.E. Lewis, V. Lewis, N. Lewis-Burke, X. Li, T. Light, L. Lightstone, W. Lilaonitkul, L. Lim, S. Linford, A. Lingford-Hughes, M. Lipman, K. Liyanage, A. Lloyd, S. Logan, D. Lomas, N.I. Lone, R. Loosley, J.M. Lord, H. Lota, W. Lovegrove, A. Lucey, E. Lukaschuk, A. Lye, C. Lynch, S. MacDonald, G. MacGowan, I. Macharia, J. Mackie, L. Macliver, S. Madathil, G. Madzamba, N. Magee, M.M. Magtoto, N. Mairs, N. Majeed, E. Major, F. Malein, M. Malim, G. Mallison, W. D-C. Man, S. Mandal, K. Mangion, C. Manisty, R. Manley, K. March, S. Marciniak, P. Marino, M. Mariveles, M. Marks, E. Marouzet, S. Marsh, B. Marshall, M. Marshall, J. Martin, A. Martineau, L.M. Martinez, N. Maskell, D. Matila, W. Matimba-Mupaya, L. Matthews, A. Mbuyisa, S. McAdoo, H. McAllister-Williams, A. McArdle, P. McArdle, D. McAulay, G.P. McCann, J. McCormick, W. McCormick, P. McCourt, L. McGarvey, C. McGee, K. Mcgee, J. McGinness, K. McGlynn, A. McGovern, H. McGuinness, I.B. McInnes, J. McIntosh, E. McIvor, K. McIvor, L. McLeavey, A. McMahon, M.J. McMahon, L. McMorrow, T. Mcnally, M. McNarry, J. McNeill, A. McQueen, H. McShane, C. Mears, C. Megson, S. Megson, P. Mehta, J. Meiring, L. Melling, M. Mencias, D. Menzies, M. Merida Morillas, A. Michael, C. Miller, L. Milligan, C. Mills, G. Mills, N.L. Mills, L. Milner, S. Misra, J. Mitchell, A. Mohamed, N. Mohamed, S. Mohammed, P.L. Molyneaux, W. Monteiro, S. Moriera, A. Morley, L. Morrison, R. Morriss, A. Morrow, A.J. Moss, P. Moss, K. Motohashi, N. Msimanga, E. Mukaetova-Ladinska, U. Munawar, J. Murira, U. Nanda, H. Nassa, M. Nasseri, A. Neal, R. Needham, P. Neill, S. Neubauer, D.E. Newby, H. Newell, T. Newman, J. Newman, A. Newton-Cox, T. Nicholson, D. Nicoll, A. Nikolaidis, C.M. Nolan, M.J. Noonan, C. Norman, P. Novotny, J. Nunag, L. Nwafor, U. Nwanguma, J. Nyaboko, C. O'Brien, K. O'Donnell, D. O'Regan, L. O'Brien, N. Odell, G. Ogg, O. Olaosebikan, C. Oliver, Z. Omar, P.J.M. Openshaw, L. Orriss-Dib, L. Osborne, R. Osbourne, M. Ostermann, C. Overton, J. Owen, J. Oxton, J. Pack, E. Pacpaco, S. Paddick, S. Painter, A. Pakzad, S. Palmer, P. Papineni, K. Paques, K. Paradowski, M. Pareek, D. Parekh, H. Parfrey, C. Pariante, S. Parker, M. Parkes, J. Parmar, S. Patale, B. Patel, M. Patel, S. Patel, D. Pattenadk, M. Pavlides, S. Payne, L. Pearce, J.E. Pearl, D. Peckham, J. Pendlebury, Y. Peng, C. Pennington, I. Peralta, E. Perkins, Z. Peterkin, T. Peto, N. Petousi, J. Petrie, P. Pfeffer, J. Phipps, J. Pimm, K. Piper Hanley, R. Pius, H. Plant, S. Plein, T. Plekhanova, M. Plowright, K. Poinasamy, O. Polgar, L. Poll, J.C. Porter, J. Porter, S. Portukhay, N. Powell, A. Prabhu, J. Pratt, A. Price, C. Price, D. Price, L. Price, A. Prickett, J. Propescu, S. Prosper, S. Pugmire, S. Quaid, J. Quigley, J. Quint, H. Qureshi, I.N. Qureshi, K. Radhakrishnan, N.M. Rahman, M. Ralser, B. Raman, A. Ramos, H. Ramos, J. Rangeley, B. Rangelov, L. Ratcliffe, P. Ravencroft, A. Reddington, R. Reddy, A. Reddy, H. Redfearn, D. Redwood, A. Reed, M. Rees, T. Rees, K. Regan, W. Reynolds, C. Ribeiro, A. Richards, E. Richardson, M. Richardson, P. Rivera-Ortega, K. Roberts, E. Robertson, E. Robinson, L. Robinson, L. Roche, C. Roddis, J. Rodger, A. Ross, G. Ross, J. Rossdale, A. Rostron, A. Rowe, A. Rowland, J. Rowland, M.J. Rowland, S.L. Rowland-Jones, K. Roy, M. Roy, I. Rudan, R. Russell, E. Russell, G. Saalmink, R. Sabit, E.K. Sage, T. Samakomva, N. Samani, C. Sampson, K. Samuel, R. Samuel, A. Sanderson, E. Sapey, D. Saralaya, J. Sargant, C. Sarginson, T. Sass, N. Sattar, K. Saunders, R.M. Saunders, P. Saunders, L.C. Saunders, H. Savill, W. Saxon, A. Sayer, J. Schronce, W. Schwaeble, J.T. Scott, K. Scott, N. Selby, M.G. Semple, M. Sereno, T.A. Sewell, A. Shah, K. Shah, P. Shah, M. Shankar-Hari, M. Sharma, C. Sharpe, M. Sharpe, S. Shashaa, A. Shaw, K. Shaw, V. Shaw, A. Sheikh, S. Shelton, L. Shenton, K. Shevket, A. Shikotra, J. Short, S. Siddique, S. Siddiqui, J. Sidebottom, L. Sigfrid, G. Simons, J. Simpson, N. Simpson, A. Singapuri, C. Singh, S. Singh, S.J. Singh, D. Sissons, J. Skeemer, K. Slack, A. Smith, D. Smith, S. Smith, J. Smith, L. Smith, M. Soares, T.S. Solano, R. Solly, A.R. Solstice, T. Soulsby, D. Southern, D. Sowter, M. Spears, L.G. Spencer, F. Speranza, L. Stadon, S. Stanel, N. Steele, M. Steiner, D. Stensel, G. Stephens, L. Stephenson, M. Stern, I. Stewart, R. Stimpson, S. Stockdale, J. Stockley, W. Stoker, R. Stone, W. Storrar, A. Storrie, K. Storton, E. Stringer, S. Strong-Sheldrake, N. Stroud, C. Subbe, C.L. Sudlow, Z. Suleiman, C. Summers, C. Summersgill, D. Sutherland, D.L. Sykes, R. Sykes, N. Talbot, A.L. Tan, L. Tarusan, V. Tavoukjian, A. Taylor, C. Taylor, J. Taylor, A. Te, H. Tedd, C.J. Tee, J. Teixeira, H. Tench, S. Terry, S. Thackray-Nocera, F. Thaivalappil, B. Thamu, D. Thickett, C. Thomas, D.C. Thomas, S. Thomas, A.K. Thomas, T. Thomas-Woods, T. Thompson, A.A.R. Thompson, T. Thornton, M. Thorpe, R.S. Thwaites, J. Tilley, N. Tinker, G.F. Tiongson, M. Tobin, J. Tomlinson, C. Tong, M. Toshner, R. Touyz, K.A. Tripp, E. Tunnicliffe, A. Turnbull, E. Turner, S. Turner, V. Turner, K. Turner, S. Turney, L. Turtle, H. Turton, J. Ugoji, R. Ugwuoke, R. Upthegrove, J. Valabhji, M. Ventura, J. Vere, C. Vickers, B. Vinson, E. Wade, P. Wade, L.V. Wain, T. Wainwright, L.O. Wajero, S. Walder, S. Walker, E. Wall, T. Wallis, S. Walmsley, J.A. Walsh, S. Walsh, L. Warburton, T.J.C. Ward, K. Warwick, H. Wassall, S. Waterson, E. Watson, L. Watson, J. Watson, J. Weir McCall, C. Welch, H. Welch, B. Welsh, S. Wessely, S. West, H. Weston, H. Wheeler, S. White, V. Whitehead, J. Whitney, S. Whittaker, B. Whittam, V. Whitworth, A. Wight, J. Wild, M. Wilkins, D. Wilkinson, B. Williams, N. Williams, J. Williams, S.A. Williams-Howard, M. Willicombe, G. Willis, J. Willoughby, A. Wilson, D. Wilson, I. Wilson, N. Window, M. Witham, R. Wolf-Roberts, C. Wood, F. Woodhead, J. Woods, D.G. Wootton, J. Wormleighton, J. Worsley, D. Wraith, C. Wrey Brown, C. Wright, L. Wright, S. Wright, J. Wyles, I. Wynter, M. Xu, N. Yasmin, S. Yasmin, T. Yates, K.P. Yip, B. Young, S. Young, A. Young, A.J. Yousuf, A. Zawia, L. Zeidan, B. Zhao, B. Zheng, O. Zongo

**Affiliations:** a Usher Institute, University of Edinburgh, Edinburgh, UK; b Blizard Institute, Barts and The London School of Medicine and Dentistry, Queen Mary University of London, London, UK; c Wolfson Institute of Population Health, Barts and The London School of Medicine and Dentistry, Queen Mary University of London, London, UK; d Department of Population Health Sciences, University of Leicester, Leicester, UK; e The Institute for Lung Health, NIHR Leicester Biomedical Research Centre, University of Leicester, Leicester, UK; f NIHR Leicester Biomedical Research Centre, University of Leicester, Leicester, UK; g University Hospitals of Leicester NHS Trust, Leicester, UK; h Centre for Exercise and Rehabilitation Science, NIHR Leicester Biomedical Research Centre-Respiratory, University of Leicester, Leicester, UK; i Therapy Department, University Hospitals of Leicester, NHS Trust, Leicester, UK; j Barts Health NHS Trust, London, UK; k Queen Mary University of London, London, UK; l UCL Respiratory, University College London, London, UK; m Centre for Inflammation Research, University of Edinburgh, Edinburgh, UK; n The Newcastle upon Tyne Hospitals NHS Foundation Trust, Newcastle, UK; o Translational and Clinical Research Institute, Newcastle University, Newcastle, UK; p Population Health Science Institute, Newcastle University, Newcastle, UK; q Centre for Medical Informatics, The Usher Institute, University of Edinburgh, Edinburgh, UK; r Royal Infirmary of Edinburgh, NHS Lothian, Edinburgh, UK; s National Heart Lung Institute, Imperial College London, London, UK; t University of Dundee, Ninewells Hospital and Medical School, Dundee, UK; u Medical Research Council (MRC) Human Immunology Unit, University of Oxford, Oxford, UK; v Division of Infection, Immunity & Respiratory Medicine, Faculty of Biology, Medicine and Health, University of Manchester, Manchester, UK; w Manchester University NHS Foundation Trust, Manchester, UK; x Department of Clinical Research, London School of Hygiene & Tropical Medicine, London, UK; y Hospital for Tropical Diseases, University College London Hospital, London, UK; z Division of Infection and Immunity, University College London, London, UK; aa Asthma and Lung UK, London, UK; ab Radcliffe Department of Medicine, University of Oxford, Oxford, UK; ac Oxford University Hospitals NHS Foundation Trust, Oxford, UK; ad Wellcome-Wolfson Institute for Experimental Medicine, Queen's University, Belfast, UK; ae Asthma UK Centre for Applied Research, Queen Mary University of London, London, UK; af Asthma UK Centre for Applied Research, University of Edinburgh, Edinburgh, UK

**Keywords:** COVID-19, Dyspnoea, Long COVID, Recovery, Cohort

## Abstract

**Background:**

The risk factors for recovery from COVID-19 dyspnoea are poorly understood. We investigated determinants of recovery from dyspnoea in adults with COVID-19 and compared these to determinants of recovery from non-COVID-19 dyspnoea.

**Methods:**

We used data from two prospective cohort studies: PHOSP-COVID (patients hospitalised between March 2020 and April 2021 with COVID-19) and COVIDENCE UK (community cohort studied over the same time period). PHOSP-COVID data were collected during hospitalisation and at 5-month and 1-year follow-up visits. COVIDENCE UK data were obtained through baseline and monthly online questionnaires. Dyspnoea was measured in both cohorts with the Medical Research Council Dyspnoea Scale. We used multivariable logistic regression to identify determinants associated with a reduction in dyspnoea between 5-month and 1-year follow-up.

**Findings:**

We included 990 PHOSP-COVID and 3309 COVIDENCE UK participants. We observed higher odds of improvement between 5-month and 1-year follow-up among PHOSP-COVID participants who were younger (odds ratio 1.02 per year, 95% CI 1.01–1.03), male (1.54, 1.16–2.04), neither obese nor severely obese (1.82, 1.06–3.13 and 4.19, 2.14–8.19, respectively), had no pre-existing anxiety or depression (1.56, 1.09–2.22) or cardiovascular disease (1.33, 1.00–1.79), and shorter hospital admission (1.01 per day, 1.00–1.02). Similar associations were found in those recovering from non-COVID-19 dyspnoea, excluding age (and length of hospital admission).

**Interpretation:**

Factors associated with dyspnoea recovery at 1-year post-discharge among patients hospitalised with COVID-19 were similar to those among community controls without COVID-19.

**Funding:**

PHOSP-COVID is supported by a grant from the MRC-UK Research and Innovation and the Department of Health and Social Care through the 10.13039/501100000272National Institute for Health Research (NIHR) rapid response panel to tackle COVID-19. The views expressed in the publication are those of the author(s) and not necessarily those of the National Health Service (NHS), the NIHR or the Department of Health and Social Care.

COVIDENCE UK is supported by the UK Research and Innovation, the National Institute for Health Research, and Barts Charity. The views expressed are those of the authors and not necessarily those of the funders.

**Research in context**

**Evidence before this study**

In a previous systematic review and meta-analysis conducted by our group, we searched PubMed and Embase for studies of post-COVID-19 dyspnoea published by November 2021, using terms related to COVID-19, long-term follow-up, and breathlessness or dyspnoea. This synthesis of 119 eligible papers showed that 20% (95% CI 15–26) of COVID-19 patients reported post-COVID-19 dyspnoea at 7–12 months post infection, with females and hospitalised/severe patients more likely to suffer from this sequela; evidence on pathophysiological mechanisms and targeted interventions was inconclusive. During the past year, more papers on long-term post-COVID-19 dyspnoea have been published. However, most of these new studies have focused on the presence of the symptom during follow-up and few have examined determinants of recovery from dyspnoea over time. Furthermore, longitudinal data on the recovery of dyspnoea in concurrent non-COVID-19 control populations are lacking for comparison.

**Added value of this study**

We used data from two nationwide prospective cohort studies of UK adults: PHOSP-COVID (one of the world's largest cohorts of post-hospitalisation COVID-19 survivors) and COVIDENCE UK (providing concurrent data from community controls on non-COVID-19 dyspnoea). Based on repeated measurements with Medical Research Council Dyspnoea Scale from PHOSP-COVID participants, we observed higher odds of improved dyspnoea from 5 months to 1 year after discharge among those who were younger, male, neither obese nor severely obese, without pre-existing depression/anxiety or cardiovascular conditions, or had shorter hospital admission. Similar associations were found among community controls with non-COVID-19 dyspnoea as measured by the same scale, excluding age (and hospitalisation duration). To our knowledge, this is one of the largest longitudinal investigations of recovery from COVID-19 dyspnoea.

**Implications of all the available evidence**

This analysis has found that determinants of dyspnoea recovery over time among patients hospitalised with COVID-19 were similar to those identified among community controls who suffered from non-COVID-19 dyspnoea. These findings imply a similar risk factor profile for the recovery of post-COVID-19 dyspnoea and non-COVID-19 dyspnoea, and provide epidemiological evidence for clinical trials assessing whether already established interventions for dyspnoea (e.g., pulmonary rehabilitation) work effectively for post-COVID-19 dyspnoea.

## Introduction

Millions of people globally continue to be infected with SARS-CoV-2 every week, but vaccination programmes and therapeutics have greatly reduced the risk of severe disease and death.^1^ With more than 650 million infections recorded throughout the pandemic,^1^ and increasing odds of surviving the disease, focus is turning to the long-term effects of COVID-19, commonly known as long COVID. Long COVID captures both ongoing symptomatic COVID-19, consisting of symptoms lasting 4–12 weeks after the acute phase, and post-COVID-19 syndrome, which describes symptoms more than 12 weeks after the original infection.^2^ The incidence and global prevalence of long COVID is unknown, but UK estimates suggest that more than 2 million people—3.5% of the population—may be affected.^3^

Dyspnoea is a common symptom of long COVID, reported by more than 40% of those affected.^3^ The symptom can be long lasting, with up to a fifth of COVID-19 survivors experiencing dyspnoea for more than 6 months after SARS-CoV-2 infection.^4^ Dyspnoea, a key predictor of quality of life and exercise tolerance, is associated with reduced functional status and worse psychological outcomes.^5^ However, the mechanisms of dyspnoea in different conditions are not yet fully understood, and management of the symptom can vary according to the underlying cause.^5^

There are a growing number of longitudinal studies assessing long COVID,^6^ and dyspnoea specifically.^4^ Our previous analysis using 5-month follow-up data of hospitalised COVID-19 patients in the PHOSP-COVID cohort showed that the risk of post-COVID-19 dyspnoea differed by a range of demographic and clinical factors.^7^ However, there remains inconsistent evidence on determinants of recovery over time. Additionally, as most studies on post-COVID-19 dyspnoea only included participants with COVID-19,^4^^,^^7^ it has not been possible to discern whether identified determinants and trajectories of recovery differ between post-COVID-19 dyspnoea and dyspnoea following other illnesses. This is of key importance both for biological understanding of the condition and for informing possible targeted interventions or rehabilitation therapies for those affected.

To address this gap, we used data from two prospective, longitudinal cohort studies to investigate determinants of recovery from dyspnoea in people hospitalised with COVID-19 comparing these to factors associated with dyspnoea from other causes in community controls.

## Methods

### Data sources

This study report adheres to STROBE (Strengthening the Reporting of Observational Studies in Epidemiology) guidelines (appendix pp 2–3).^8^ We analysed data from two prospective cohort studies of UK adults: PHOSP-COVID and COVIDENCE UK.

PHOSP-COVID is a multicentre study of adults (18 years or older) who were discharged following inpatient treatment for COVID-19 from one of 83 National Health Service (NHS) hospitals in the UK. In PHOSP-COVID, COVID-19 status was ascertained by a reverse transcriptase polymerase chain reaction (RT-PCR) test for SARS-CoV-2 or a clinician diagnosis. Individuals were excluded if they attended the emergency department but were not admitted to hospital or if they had an existing condition with a life expectancy of less than 6 months. For this analysis, we used data on participants discharged from hospital between March 2020 and April 2021. Further details of this cohort have been described previously.^9^

Data from COVIDENCE UK were analysed with the main aim to assess determinants of dyspnoea recovery among control participants without COVID-19. COVIDENCE UK is a longitudinal, population-based observational study of COVID-19 in UK residents aged 16 years or older.^10^ Participants were invited via a national media campaign to complete an online baseline questionnaire and monthly follow-up questionnaires capturing information on potential symptoms of COVID-19, results of nose or throat swab tests for SARS-CoV-2, COVID-19 vaccination status, medical history, and ongoing symptoms including dyspnoea. The study was launched on May 1, 2020, and closed to enrolment on October 6, 2021.

### Ethical approval and registration

PHOSP-COVID was approved by the Leeds West Research Ethics Committee (20/YH/0225) and is registered with the ISRCTN Registry (ISRCTN10980107). COVIDENCE UK was approved by Leicester South Research Ethics Committee (ref 20/EM/0117) and is registered with ClinicalTrials.gov (NCT04330599). All participants in both studies provided informed consent to participate.

### Procedures and study participants

#### PHOSP-COVID cohort

In PHOSP-COVID, participant demographics and clinical characteristics were obtained from hospital notes by the study team at each site or during the first in-person research visit. Participants were invited to attend research visits at 2–7 months (5-month visit) and at 10–14 months (1-year visit) after discharge. At each of these visits, participants were asked to report their breathlessness level using the Medical Research Council (MRC) Dyspnoea Scale,^11^ a validated and widely used five-point scale for grading the effect of dyspnoea on daily activities. Scores ranged from "not troubled by breathlessness except on strenuous exercise" (score 1) to "too breathless to leave the house, or breathless when dressing/undressing" (score 5). At the 5-month visit, participants were additionally asked to recall their level of breathlessness before COVID-19 onset using the same scale, thus providing a recalled baseline value.

For this study, we included the subset of PHOSP-COVID participants who attended both 5-month and 1-year in-person visits after discharge. Participants with missing data in age, sex, or ethnicity (n = 7) were excluded from analysis.

#### COVIDENCE UK cohort

Demographic and clinical characteristics for COVIDENCE UK participants were obtained from their baseline questionnaire, along with their perceived breathlessness as reported with the MRC Dyspnoea Scale. We extracted follow-up dyspnoea scores from the monthly questionnaires completed most closely to 5 months and 1 year after start of follow-up; to align with PHOSP-COVID, we included questionnaires completed between 2 and 7 months (for the 5-month visit) and 10 and 14 months (for the 1-year visit) after start of follow-up.

For this analysis, we categorised COVIDENCE UK participants as COVID-19 cases (any participants who reported a positive SARS-CoV-2 test or doctor-diagnosed or hospital-diagnosed COVID-19 at baseline or during follow-up) or controls without COVID-19 (included in the main analysis). We included all participants with at least 10 months’ follow-up after baseline survey (controls) or date of SARS-CoV-2 infection (cases), and excluded control participants who reported probable COVID-19 symptoms (as calculated from a combination of self-reported symptoms^12^) or suspected long COVID at baseline or during follow-up.

Participants in both cohorts were assigned Index of Multiple Deprivation (IMD) 2019 scores, or equivalent scores for devolved nations, according to their postcode.

### Outcomes

As mentioned, the PHOSP-COVID cohort provided data on participants’ dyspnoea level at three timepoints (5-month visit, 1-year visit, and recalled pre-COVID level at 5-month visit), but due to concerns of possible recall bias, we pre-specified the improvement of dyspnoea based on 1-year *vs* 5-month data as the main outcome, and the status based on 1-year *vs* recalled pre-COVID data (i.e., baseline data) as an exploratory outcome. We applied a similar strategy to COVIDENCE UK controls.

Specifically, the primary outcome was participant-reported improvement in dyspnoea between 5-month and 1-year follow-up, defined as a reduction (at least by 1 point) in the MRC Dyspnoea score between the two timepoints. Participants who did not report an improvement were classified as having worse or persistent dyspnoea. For the analysis of this main outcome, participants who reported an MRC Dyspnoea score of 1 at the 5-month visit were excluded, as they had no possibility of further recovery (i.e., our target population was those with dyspnoea symptoms at 5-month visit).

When compared with baseline data (which refers to recalled pre-COVID-19 data for PHOSP-COVID), a lower MRC Dyspnoea score at the 1-year visit no longer reflects recovery; indeed, defining recovery based on these two timepoints was difficult, as participants could have experienced no dyspnoea symptoms throughout the interval, and thus could not be classified as having “recovered”. Therefore, the exploratory outcome variable we defined based on the 1-year and baseline MRC Dyspnoea scores was long-term post-COVID-19 dyspnoea (i.e., worsening dyspnoea over 1 year). PHOSP-COVID participants whose 1-year score was higher than their recalled pre-COVID-19 score were classified as having long-term post-COVID-19 dyspnoea, whereas those whose 1-year score was equal to or lower than their recalled pre-COVID-19 score were classified as not having long-term post-COVID-19 dyspnoea. Among COVIDENCE UK participants, scores from the baseline survey and 1-year survey were similarly compared, to assess whether they had worsening dyspnoea over 1 year. Participants who had the highest degree of dyspnoea at baseline (i.e., score = 5) were excluded. The analysis of this exploratory outcome aimed to answer a different question: which subsets of population were more likely to experience long-term dyspnoea 1 year after COVID hospitalisation.

### Statistical analysis

For the primary analysis, we used univariable and multivariable logistic regression to assess potential determinants of improved dyspnoea (*vs* persistent or worse dyspnoea) between 5-month and 1-year follow-up in the PHOSP-COVID cohort and COVIDENCE UK controls separately, including age, sex, ethnicity, IMD 2019 quintile, body mass index (BMI), smoking status, pre-existing cardiovascular or respiratory diseases, and pre-existing anxiety or depression as covariates. For PHOSP-COVID, we did a further analysis including the following hospitalisation characteristics as predictors: length of hospital stay, level of respiratory support received (categorised according to the World Health Organization [WHO] Clinical Progression Scale^13^), pulmonary embolism, proning, and use of antibiotics, systemic steroids, or anticoagulants during hospitalisation. Missing values of predictors were treated as a separate category. For the multivariable regressions, we used backwards stepwise selection to determine the final model, with an elimination criterion of p > 0.1. In all multivariable analyses, we adjusted for 5-month dyspnoea score.

We conducted several sensitivity analyses to assess the robustness of the main findings: (1) using a fully adjusted logistic regression model (i.e., including all covariates without backwards selection); (2) repeating the final model using complete-case analysis or (3) multiple imputation by chained equations to deal with missing data; (4) excluding participants with 5-month dyspnoea score equal to or lower than the baseline or pre-COVID-19 score; (5) assessing the non-linear effect of age and length of hospital stay by including squared terms; and (6) additionally adjusting for calendar date of hospital admission and time interval between research visits using restricted cubic splines with five knots.

We also carried out several pre-planned exploratory analyses, using similar analytical procedures. We first repeated the primary analysis in COVIDENCE UK cases. For COVIDENCE UK cases, follow-up began at the date of the first positive SARS-CoV-2 test or diagnosis of COVID-19. We then repeated the primary analysis in all COVIDENCE UK participants, including COVID-19 case status as a potential determinant and exploring interactions between COVID-19 case status and all other covariates retained in the final model. Finally, we analysed potential determinants of long-term dyspnoea in both PHOSP-COVID cases and COVIDENCE UK controls, including any participants with baseline (recalled, for PHOSP-COVID) and 1-year MRC Dyspnoea scores; we excluded COVIDENCE UK participants who reported definite or probable COVID-19 at baseline, as their baseline score would have been provided after COVID-19 onset. In these analyses, we adjusted for baseline or pre-COVID-19 dyspnoea scores.

All statistical tests were two-sided, with p < 0.05 set as the significance threshold. We used Stata (versions 15.0 and 17.0) for all analyses.

### Role of the funding source

The funders of PHOSP-COVID and COVIDENCE UK had no role in study design, data collection, data analysis, data interpretation, or writing of the report.

## Results

**Fig. 1:**
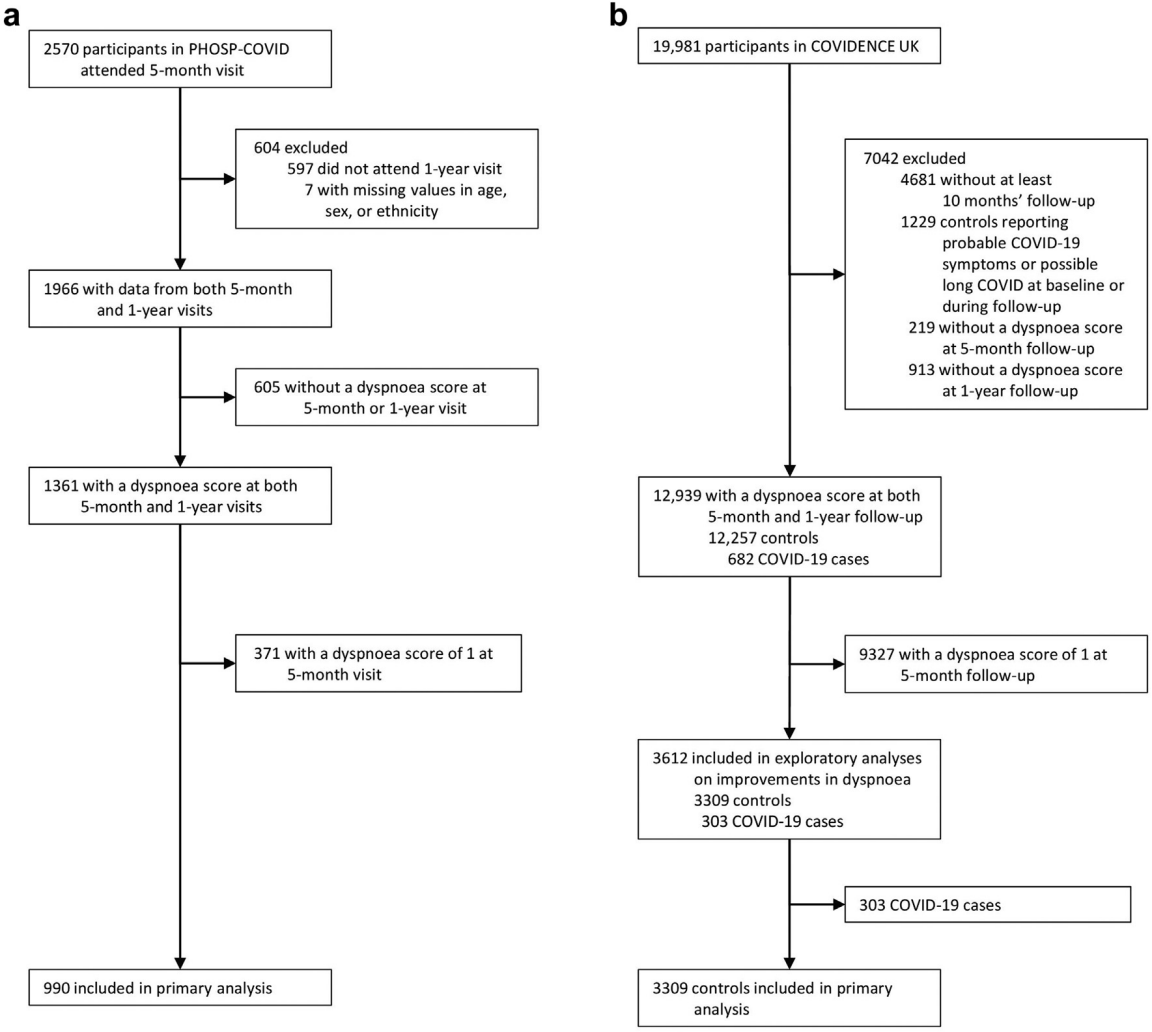
**Cohort profiles for PHOSP-COVID (a) and COVIDENCE UK (b)**.

Among PHOSP-COVID participants who attended both the 5-month and 1-year research visits, 2082 completed the MRC Dyspnoea Scale at their 5-month visit (median score 2 [IQR 1–4]), 1703 completed it at their 1-year visit (median score 2 [1–4]), and 1890 recalled their pre-COVID-19 level (median score 1 [1–2]). Among the 1361 participants with both 5-month and 1-year dyspnoea scores, 371 (27.3%) reported an MRC Dyspnoea score of 1 at their 5-month visit, and the remaining 990 (72.7%) were included in the primary analysis (Fig. 1a). These participants were discharged from hospital between March 7, 2020, and April 13, 2021.

Among 12,939 COVIDENCE UK participants with both 5-month and 1-year dyspnoea scores, 9327 (72.1%) reported an MRC Dyspnoea score of 1 at 5-month follow-up and 303 (2.3%) were confirmed COVID-19 cases, leaving 3309 (25.6%) participants to be included as dyspnoea controls in the primary analysis (Fig. 1b). Start of follow-up for these participants ranged from May 1, 2020, to May 27, 2021.

Baseline characteristics varied between the two cohorts, with PHOSP-COVID participants more likely to be male, non-White, socioeconomically deprived, overweight or obese, and have cardiovascular disease (Table 1). PHOSP-COVID participants were also more likely to have worse dyspnoea at 5 months than COVIDENCE UK dyspnoea controls (Table 1).

**Table 1:** Participant characteristics by recovery status of dyspnoea symptoms from 5 months to 1 year after enrolment.

	PHOSP-COVID (hospitalised COVID-19 cases)			COVIDENCE UK (community controls)		
	All participants (n = 990)	Improved dyspnoea (n = 369)	Worse or persistent dyspnoea (n = 621)	All participants (n = 3309)	Improved dyspnoea (n = 915)	Worse or persistent dyspnoea (n = 2394)
**Sociodemographics**						
Age, years	59.1 (11.9)	57.9 (12.1)	59.9 (11.6)	61.1 (13.1)	61.0 (12.5)	61.2 (13.3)
Sex						
Female	443 (44.7%)	151 (40.9%)	292 (47.0%)	2555 (77.2%)	672 (73.4%)	1883 (78.7%)
Male	547 (55.3%)	218 (59.1%)	329 (53.0%)	754 (22.8%)	243 (26.6%)	511 (21.3%)
Ethnicity						
White	760 (76.8%)	276 (74.8%)	484 (77.9%)	3167 (95.7%)	877 (95.8%)	2290 (95.7%)
Black	70 (7.1%)	29 (7.9%)	41 (6.6%)	15 (0.5%)	6 (0.7%)	9 (0.4%)
South Asian	98 (9.9%)	40 (10.8%)	58 (9.3%)	38 (1.1%)	7 (0.8%)	31 (1.3%)
Mixed	23 (2.3%)	9 (2.4%)	14 (2.3%)	54 (1.6%)	14 (1.5%)	40 (1.7%)
Other	39 (3.9%)	15 (4.1%)	24 (3.9%)	35 (1.1%)	11 (1.2%)	24 (1.0%)
IMD quintile						
1 (least deprived)	172/987 (17.4%)	70/368 (19.0%)	102/619 (16.5%)	935 (28.3%)	280 (30.6%)	655 (27.4%)
2	169/987 (17.1%)	63/368 (17.1%)	106/619 (17.1%)	855 (25.8%)	245 (26.8%)	610 (25.5%)
3	182/987 (18.4%)	64/368 (17.4%)	118/619 (19.1%)	734 (22.2%)	195 (21.3%)	539 (22.5%)
4	230/987 (23.3%)	85/368 (23.1%)	145/619 (23.4%)	520 (15.7%)	132 (14.4%)	388 (16.2%)
5 (most deprived)	234/987 (23.7%)	86/368 (23.4%)	148/619 (23.9%)	265 (8.0%)	63 (6.9%)	202 (8.4%)
**Clinical characteristics**						
BMI, kg/m^2^	32.5 (6.7)	31.8 (6.6)	32.8 (6.8)	28.7 (6.3)	28.0 (6.1)	29.0 (6.4)
<25 (normal or underweight)	70/692 (10.1%)	34/255 (13.3%)	36/437 (8.2%)	1031/3302 (31.2%)	321 (35.1%)	710/2387 (29.7%)
25 to <30 (overweight)	206/692 (29.8%)	81/255 (31.8%)	125/437 (28.6%)	1106/3302 (33.5%)	326 (35.6%)	780/2387 (32.7%)
30 to <40 (obese)	330/692 (47.7%)	115/255 (45.1%)	215/437 (49.2%)	990/3302 (30.0%)	226 (24.7%)	764/2387 (32.0%)
≥40 (severely obese)	86/692 (12.4%)	25/255 (9.8%)	61/437 (14.0%)	175/3302 (5.3%)	42 (4.6%)	133/2387 (5.6%)
Current or ex-smoker	433/916 (47.3%)	148/337 (43.9%)	285/579 (49.2%)	1631 (49.3%)	445 (48.6%)	1186/2387 (49.5%)
Comorbidity						
Cardiovascular disease	484 (48.9%)	162 (43.9%)	322 (51.9%)	210 (6.3%)	47 (5.1%)	163 (6.8%)
Respiratory disease	292 (29.5%)	112 (30.4%)	180 (29.0%)	784 (23.7%)	209 (22.8%)	575 (24.0%)
Depression or anxiety	195 (19.7%)	62 (16.8%)	133 (21.4%)	1165/3307 (35.2%)	302 (33.0%)	863/2392 (36.1%)
MRC Dyspnoea score at 5 months^a^						
2	345 (34.9%)	114 (30.9%)	231 (37.2%)	2881 (87.1%)	742 (81.1%)	2139 (89.3%)
3	305 (30.8%)	111 (30.1%)	194 (31.2%)	341 (10.3%)	130 (14.2%)	211 (8.8%)
4	207 (20.9%)	81 (21.9%)	126 (20.3%)	81 (2.4%)	40 (4.4%)	41 (1.7%)
5	133 (13.4%)	63 (17.1%)	70 (11.3%)	6 (0.2%)	3 (0.3%)	3 (0.1%)
**Hospitalisation characteristics**						
Length of hospital stay, days	8 (4–17)	8 (4–15)	8 (4–19)	..	..	..
WHO clinical progression scale				..	..	..
WHO – class 3–4	149 (15.1%)	56 (15.2%)	93 (15.0%)	..	..	..
WHO – class 5	420 (42.4%)	155 (42.0%)	265 (42.7%)	..	..	..
WHO – class 6	230 (23.2%)	95 (25.7%)	135 (21.7%)	..	..	..
WHO – class 7–9	191 (19.3%)	63 (17.1%)	128 (20.6%)	..	..	..
Pulmonary embolism during hospitalisation	96/950 (10.1%)	41/349 (11.7%)	55/601 (9.2%)	..	..	..
Treatment during hospitalisation				..	..	..
Proning	179/891 (20.1%)	66/326 (20.2%)	113/565 (20.0%)	..	..	..
Antibiotic therapy	751/969 (77.5%)	271/359 (75.5%)	480/610 (78.7%)	..	..	..
Systemic (oral or IV) steroids	547/944 (57.9%)	212/352 (60.2%)	335/592 (56.6%)	..	..	..
Therapeutic dose anticoagulation	437/935 (46.7%)	170/350 (48.6%)	267/585 (45.6%)	..	..	..

Data are n (%), mean (SD), or median (IQR). BMI = body-mass index. IMD = Index of Multiple Deprivation. IV = intravenous. MRC = Medical Research Council. WHO = World Health Organization.

a Participants with no dyspnoea symptoms (MRC score = 1) at 5 months were excluded from this analysis.

**Fig. 2:**
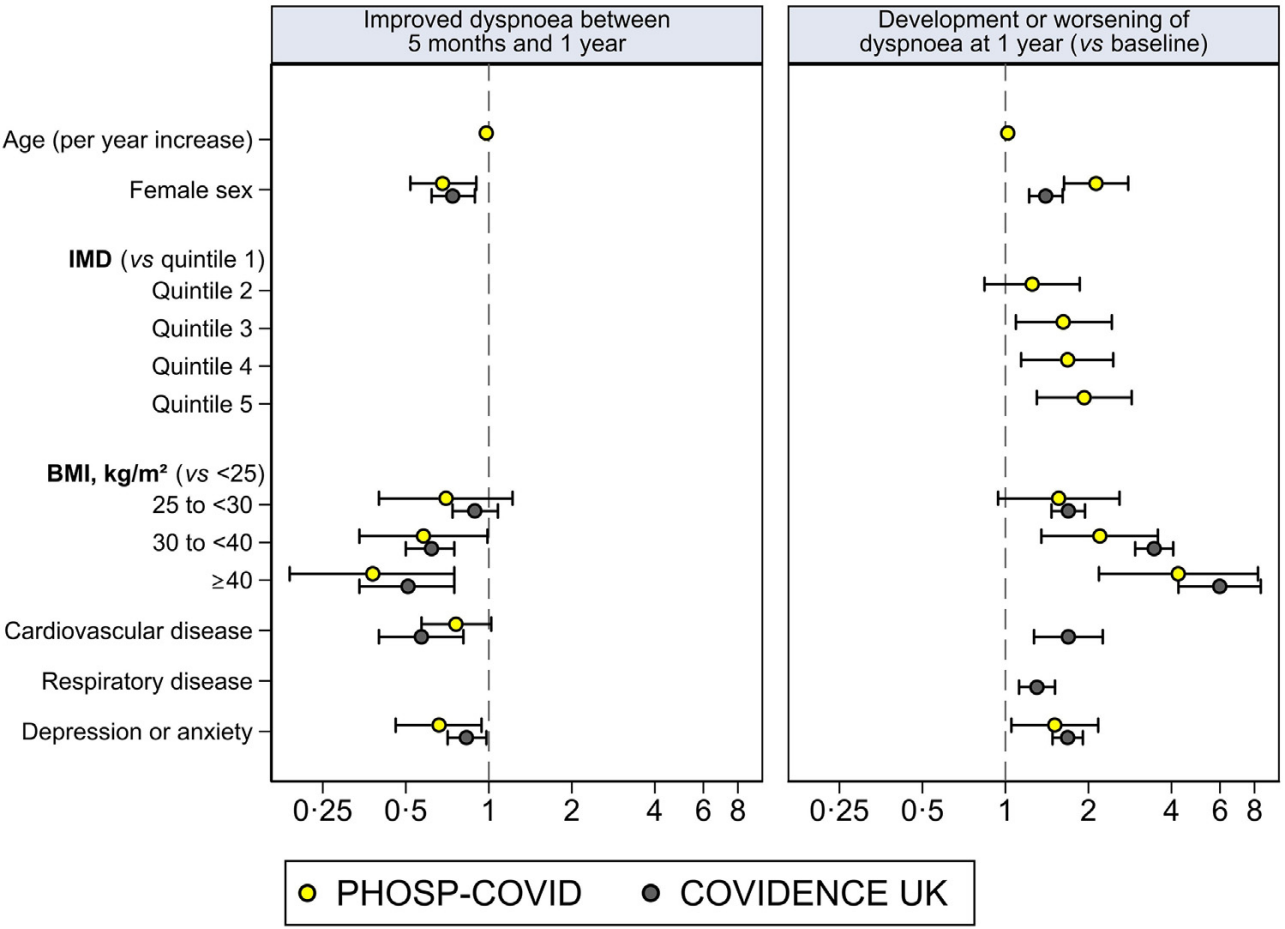
**Odds ratios (95% CIs) for observed predictors in PHOSP-COVID and COVIDENCE UK. Odds ratios are shown for significant predictors included in both cohorts**.

Among the 990 PHOSP-COVID participants with dyspnoea at 5-month follow-up, 369 (37.3%) had improved dyspnoea at 1-year follow-up, and 621 (62.7%) had the same level or worse dyspnoea (n = 419 and n = 202, respectively). We observed reduced odds of improved dyspnoea from 5 months to 1 year after discharge among patients hospitalised with COVID-19 who were older, female, obese or severely obese, and with pre-existing depression or anxiety (Table 2; Fig. 2). When including hospitalisation variables, we additionally found reduced odds of improved dyspnoea among patients with longer hospital stays and pre-existing cardiovascular conditions, whilst the associations for other predictors as observed in the previous model remained similar (Table 2). Higher MRC Dyspnoea score at 5 months was positively associated with the likelihood of improved dyspnoea at 1 year (Table 2). Results in the COVIDENCE UK control cohort were similar, with the exception of age (no association found; Table 2; Fig. 2). Results of sensitivity analyses in the PHOSP-COVID cohort were consistent with the main findings (appendix Table S1), and no significant non-linear effect was detected for age or duration of hospital stay. Results of sensitivity analyses in the COVIDENCE UK control cohort were largely consistent, with the fully adjusted model showing reduced odds of improvement among older participants (OR 0.99 per year, 95% CI 0.99–1.00) and participants residing in the most deprived IMD quintile (0.70, 0.51–0.97; appendix Table S2).

**Table 2:** Associations between participant characteristics and improvement of dyspnoea symptoms from 5 months to 1 year.

	PHOSP-COVID hospitalised COVID-19 cases (n = 990)			COVIDENCE UK community controls (n = 3309)	
	Univariable	Multivariable (including hospitalisation variables)	Multivariable (excluding hospitalisation variables)	Univariable	Multivariable
Age, per year	0.99 (0.98–1.00)	0.98 (0.97–0.99)	0.98 (0.97–0.99)	1.00 (0.99–1.00)	..
Sex					
Female	0.78 (0.60–1.01)	0.65 (0.49–0.86)	0.68 (0.52–0.90)	0.75 (0.63–0.90)	0.74 (0.62–0.89)
Male	1 (ref)	1 (ref)	1 (ref)	1 (ref)	1 (ref)
Ethnicity					
White	1 (ref)	..	..	1 (ref)	..
Black	1.24 (0.75–2.04)	..	..	1.74 (0.62–4.90)	..
South Asian	1.21 (0.79–1.86)	..	..	0.59 (0.26–1.34)	..
Mixed	1.13 (0.48–2.64)	..	..	0.91 (0.49–1.69)	..
Other	1.10 (0.57–2.12)	..	..	1.20 (0.58–2.45)	..
IMD quintile					
1 (least deprived)	1 (ref)	..	..	1 (ref)	..
2	0.87 (0.56–1.34)	..	..	0.94 (0.77–1.15)	..
3	0.79 (0.51–1.22)	..	..	0.85 (0.68–1.05)	..
4	0.85 (0.57–1.28)	..	..	0.80 (0.62–1.01)	..
5 (most deprived)	0.85 (0.57–1.27)	..	..	0.73 (0.53–1.00)	..
BMI, kg/m^2^					
<25 (normal or underweight)	1 (ref)	1 (ref)	1 (ref)	1 (ref)	1 (ref)
25 to <30 (overweight)	0.69 (0.40–1.18)	0.67 (0.38–1.17)	0.70 (0.40–1.22)	0.92 (0.77–1.11)	0.89 (0.74–1.08)
30 to <40 (obese)	0.57 (0.34–0.95)	0.55 (0.32–0.94)	0.58 (0.34–0.99)	0.65 (0.54–0.80)	0.62 (0.50–0.75)
≥40 (severely obese)	0.43 (0.22–0.84)	0.36 (0.18–0.72)	0.38 (0.19–0.75)	0.70 (0.48–1.01)	0.51 (0.34–0.75)
Current or ex-smoker	0.81 (0.62–1.06)	..	..	0.96 (0.83–1.12)	..
Comorbidity					
Cardiovascular disease	0.73 (0.56–0.94)	0.75 (0.56–1.00)	0.76 (0.57–1.02)	0.74 (0.53–1.03)	0.57 (0.40–0.81)
Respiratory disease	1.07 (0.81–1.41)	..	..	0.94 (0.78–1.12)	..
Depression or anxiety	0.74 (0.53–1.03)	0.64 (0.45–0.92)	0.66 (0.46–0.94)	0.87 (0.74–1.03)	0.83 (0.71–0.98)
Length of hospital stay, per day	0.99 (0.99–1.00)	0.99 (0.98–1.00)	..	..	..
WHO clinical progression scale					
WHO – class 3–4	1 (ref)	..	..	..	..
WHO – class 5	0.97 (0.66–1.43)	..	..	..	..
WHO – class 6	1.17 (0.77–1.78)	..	..	..	..
WHO – class 7–9	0.82 (0.52–1.28)	..	..	..	..
Pulmonary embolism during hospitalisation	1.32 (0.86–2.03)	..	..	..	..
Treatment during hospitalisation					
Proning	1.02 (0.72–1.43)	..	..	..	..
Antibiotic therapy	0.83 (0.61–1.14)	..	..	..	..
Systemic (oral or IV) steroids	1.16 (0.89–1.52)	..	..	..	..
Therapeutic dose anticoagulation	1.12 (0.86–1.47)	..	..	..	..
MRC Dyspnoea score at 5 months^a^					
2	1 (ref)	1 (ref)	1 (ref)	1 (ref)	1 (ref)
3	1.16 (0.84–1.60)	1.33 (0.95–1.85)	1.29 (0.92–1.79)	1.78 (1.41–2.24)	2.09 (1.64–2.68)
4	1.30 (0.91–1.86)	1.77 (1.21–2.60)	1.70 (1.16–2.48)	2.81 (1.80–4.38)	3.79 (2.37–6.05)
5	1.82 (1.21–2.74)	2.57 (1.66–3.99)	2.37 (1.54–3.64)	2.88 (0.58–14.31)	3.37 (0.66–17.08)

Data are odds ratio (95% CI), with worse or persistent dyspnoea as the reference category. BMI = body-mass index. IMD=Index of Multiple Deprivation. IV = intravenous. MRC = Medical Research Council. WHO = World Health Organization.

a Participants with no dyspnoea symptoms (MRC score = 1) at 5 months were excluded from this analysis.

Exploratory analyses in the 303 COVIDENCE UK cases who reported having COVID-19 were underpowered, but nonetheless showed reduced odds of improved dyspnoea from 5 months to 1 year after COVID-19 onset in older (OR 0.97 per year, 95% CI 0.95–0.99) and female (0.44, 0.23–0.83; appendix Table S4) participants. We found no evidence that COVID-19 case status affected the odds of improved dyspnoea in the 3612 COVIDENCE UK participants, and no significant interactions between COVID-19 case status and other predictors were retained in the final model (appendix Table S4). This suggests that the trajectory and risk factors for dyspnoea recovery are similar between COVID-19 cases and non-COVID-19 controls within COVIDENCE UK.

Among 1188 PHOSP-COVID participants with both recalled pre-COVID-19 and 1-year dyspnoea scores (appendix Fig. S1), 37 (3.1%) had a dyspnoea score of 5 before COVID-19, and the remaining 1151 (96.9%) were included in the exploratory analysis of long-term post-COVID-19 dyspnoea. Of the 12,967 COVIDENCE UK participants with both baseline and 1-year dyspnoea scores (appendix Fig. S1), 11 (<0.1%) had a baseline dyspnoea score of 5 and 548 (4.2%) were confirmed COVID-19 cases, leaving 12,408 (95.7%) participants to be included as controls in the long-term dyspnoea exploratory analysis. Baseline characteristics for the two cohorts by long-term dyspnoea status are shown in the appendix (Table S5).

We observed increased odds of long-term post-COVID-19 dyspnoea among patients hospitalised with COVID-19 who were older, female, more deprived, obese or severely obese, ex or current smokers, and with pre-existing depression or anxiety (Table 3, Fig. 2). When including hospitalisation variables, we additionally found increased odds of long-term post-COVID-19 dyspnoea among patients with longer hospital stays (Table 3). A higher MRC Dyspnoea score before COVID-19 was negatively associated with the likelihood of having long-term post-COVID-19 dyspnoea. In the COVIDENCE UK control cohort, we also observed increased odds of worsening dyspnoea over 1 year in participants who were female; overweight, obese, or severely obese; current or ex smokers; and with pre-existing depression or anxiety; as well as among those with cardiovascular or respiratory disease (Table 3, Fig. 2).

**Table 3:** Associations between participants characteristics and development or worsening of dyspnoea symptoms at 1 year.

	PHOSP-COVID hospitalised COVID-19 cases (n = 1151)			COVIDENCE UK community controls (n = 12,408)	
	Univariable	Multivariable (including hospitalisation variables)	Multivariable (excluding hospitalisation variables)	Univariable	Multivariable
Age, per year	1.00 (0.99–1.01)	1.01 (1.00–1.03)	1.02 (1.00–1.03)	1.00 (0.99–1.00)	..
Sex					
Female	1.71 (1.34–2.17)	2.28 (1.73–2.99)	2.13 (1.63–2.79)	1.23 (1.08–1.40)	1.40 (1.22–1.61)
Male	1 (ref)	1 (ref)	1 (ref)	1 (ref)	1 (ref)
Ethnicity					
White	1 (ref)	..	..	1 (ref)	..
Black	1.26 (0.79–2.01)	..	..	1.11 (0.50–2.45)	..
South Asian	0.94 (0.64–1.38)	..	..	1.17 (0.74–1.86)	..
Mixed	0.66 (0.30–1.46)	..	..	1.57 (1.02–2.42)	..
Other	1.54 (0.84–2.85)	..	..	0.70 (0.37–1.34)	..
IMD quintile					
1 (least deprived)	1 (ref)	1 (ref)	1 (ref)	1 (ref)	..
2	1.21 (0.83–1.76)	1.26 (0.84–1.89)	1.25 (0.84–1.86)	1.04 (0.90–1.21)	..
3	1.57 (1.08–2.29)	1.68 (1.12–2.52)	1.62 (1.09–2.43)	1.09 (0.93–1.28)	..
4	1.53 (1.07–2.19)	1.73 (1.18–2.55)	1.68 (1.14–2.46)	1.15 (0.96–1.38)	..
5 (most deprived)	1.83 (1.27–2.64)	1.93 (1.29–2.87)	1.93 (1.30–2.87)	1.27 (1.01–1.61)	..
BMI, kg/m^2^					
<25 (normal or underweight)	1 (ref)	1 (ref)	1 (ref)	1 (ref)	1 (ref)
25 to <30 (overweight)	1.45 (0.90–2.34)	1.53 (0.91–2.57)	1.56 (0.94–2.59)	1.48 (1.29–1.69)	1.69 (1.47–1.94)
30 to <40 (obese)	2.00 (1.26–3.15)	2.22 (1.35–3.64)	2.20 (1.35–3.57)	2.34 (2.02–2.72)	3.46 (2.95–4.06)
≥40 (severely obese)	3.14 (1.70–5.79)	4.19 (2.14–8.19)	4.23 (2.18–8.23)	3.01 (2.20–4.13)	5.98 (4.24–8.43)
Current or ex-smoker	1.38 (1.08–1.77)	1.59 (1.21–2.09)	1.58 (1.21–2.07)	1.35 (1.20–1.51)	1.35 (1.20–1.52)
Comorbidity					
Cardiovascular disease	1.05 (0.83–1.32)	..	..	1.33 (1.01–1.74)	1.69 (1.27–2.25)
Respiratory disease	0.79 (0.61–1.04)	..	..	1.23 (1.06–1.42)	1.30 (1.12–1.51)
Depression or anxiety	1.33 (0.96–1.85)	1.52 (1.05–2.20)	1.51 (1.05–2.17)	1.54 (1.36–1.74)	1.68 (1.48–1.91)
Length of hospital stay, per day	1.02 (1.01–1.03)	1.02 (1.01–1.03)	..	..	..
WHO clinical progression scale					
WHO – class 3–4	1 (ref)	..	..	..	..
WHO – class 5	0.81 (0.57–1.16)	..	..	..	..
WHO – class 6	0.98 (0.66–1.44)	..	..	..	..
WHO – class 7–9	1.81 (1.19–2.74)	..	..	..	..
Pulmonary embolism during hospitalisation	1.15 (0.78–1.70)	..	..	..	..
Treatment during hospitalisation					
Proning	1.30 (0.97–1.76)	..	..	..	..
Antibiotic therapy	1.12 (0.84–1.49)	..	..	..	..
Systemic (oral or IV) steroids	0.94 (0.74–1.20)	..	..	..	..
Therapeutic dose anticoagulation	1.02 (0.80–1.29)	..	..	..	..
MRC Dyspnoea score before COVID-19 or at baseline^a^					
1	1 (ref)	1 (ref)	1 (ref)	1 (ref)	1 (ref)
2	0.66 (0.48–0.90)	0.52 (0.37–0.72)	0.51 (0.36–0.71)	0.42 (0.36–0.50)	0.24 (0.20–0.29)
3	0.37 (0.25–0.53)	0.22 (0.15–0.33)	0.22 (0.15–0.33)	0.54 (0.34–0.84)	0.20 (0.12–0.32)
4	0.32 (0.19–0.54)	0.18 (0.10–0.31)	0.17 (0.09–0.30)	0.28 (0.09–0.88)	0.09 (0.03–0.30)

Data are odds ratio (95% CI), with stable or improved dyspnoea as the reference category. Missing values for IMD (n < 5), smoking status (n = 94), BMI (n = 379), pulmonary embolism (n = 53), and the use of proning (n = 123), antibiotics (n = 36), systemic steroids (n = 61), and therapeutic dose anticoagulation (n = 65) in the PHOSP dataset and for depression and anxiety (n = 8) were treated as modelled as separate categories. BMI = body-mass index. IMD = Index of Multiple Deprivation. IV = intravenous. MRC = Medical Research Council. WHO = World Health Organization.

a Recalled pre-COVID-19 score for PHOSP-COVID participants and baseline score for COVIDENCE UK participants. Participants with the highest degree of dyspnoea (MRC score = 5) at study entry were excluded from this analysis.

## Discussion

In this analysis of a large, prospective, observational study of patients hospitalised with COVID-19, we found that older age, female sex, obesity, pre-existing depression or anxiety, or cardiovascular disease, and increasing length of hospital stay reduced the likelihood of improvement in long-term dyspnoea. By comparing with a control population with non-COVID-19 dyspnoea, we found that most of these factors, excluding age (and length of hospital admission), were similarly negatively associated with improvements in non-COVID-19 dyspnoea in the community.

Understanding the risk factors for persistent post-COVID-19 dyspnoea not only enables targeted interventions for high-risk individuals, but could also provide insights into underlying mechanisms and management of symptoms—particularly when the risk factors are shared by people with persistent non-COVID-19 dyspnoea. Female sex, which was one of our strongest predictors of persistent, long-term dyspnoea across both hospitalised COVID-19 cases and community controls, has previously been shown to increase risk of dyspnoea in people with COVID-19^14^^,^^15^ and in the general population.^16^ In population studies, sex differences in dyspnoea prevalence have been linked to smaller lung size in female individuals,^17^^,^^18^ suggesting that reduced spirometric lung volumes could contribute to post-COVID-19 dyspnoea. We also found obesity was consistently predictive of persistent, long-term dyspnoea in both COVID-19 cases and community controls, in line with previous studies.^15^^,^^19^ Obesity can affect lung function through various mechanisms including deconditioning,^20^ and weight loss has been shown to improve pulmonary function parameters in people with obesity,^20^ and hence is a potential approach to improving post-COVID-19 dyspnoea in these individuals.

We found that pre-existing anxiety and depression consistently predicted increased risk of long-term dyspnoea and reduced the likelihood of the symptom improving between 5-month and 1-year follow-up. This is supported by previous studies that demonstrated an increased risk of long COVID symptoms among participants with pre-existing mental health disorders.^21^^,^^22^ The mechanism responsible for this effect remains unclear, particularly given the potentially bidirectional relationship between mental health problems and respiratory disease.^23^ Chronic dyspnoea has been associated with both anxiety and depression,^24^ and the severity of dyspnoea may be exacerbated by these conditions.^24^^,^^25^ However, there is little evidence on whether treatment of underlying mental health disorders can improve dyspnoea,^25^ and trials are needed to evaluate treatment of anxiety and depression as a potential pathway to alleviating dyspnoea.

While predictors of dyspnoea outcomes were largely similar in patients hospitalised with COVID-19 and community controls, we found that age was a significant predictor only among hospitalised patients. The role of age in post-COVID-19 dyspnoea is poorly understood, with existing studies reporting conflicting results.^4^ Our finding that younger participants had increased odds of improvement in long-term symptoms, among both hospitalised and community COVID-19 cases, suggests that age can indeed affect recovery. We also found older age to be a predictor of long-term post-COVID-19 dyspnoea in hospitalised patients, with reduced odds of improvement for every year increase in age. In contrast, older age did not predict worsening of dyspnoea over 1 year in community controls, highlighting the rapid and long-lasting increase in breathlessness that can be experienced by older people with COVID-19.

Cardiovascular disease is a risk factor for severe COVID-19^26^ that is itself associated with dyspnoea,^27^ as shown by the high prevalence of cardiovascular disease among PHOSP-COVID participants, and the associations found in our analyses of COVIDENCE UK controls. However, pre-existing cardiovascular disease was not found to affect the risk of long-term post-COVID-19 dyspnoea among patients hospitalised with COVID-19. The picture is further complicated by long-term effects of COVID-19 on cardiovascular health, with increased 1-year risk and burden of cardiovascular disease among COVID-19 survivors.^28^ It is therefore possible that some of the burden of post-COVID-19 dyspnoea is due to cardiovascular disorders that develop after the acute COVID-19 phase, and assessment for cardiovascular causes should therefore be included when patients with long COVID present with long-term dyspnoea.

In hospitalised patients, we found longer hospital stay—at least in part a marker of COVID-19 severity and rapid deconditioning—to be negatively associated with improved dyspnoea between 5 months and 1 year, and positively associated with long-term post-COVID-19 dyspnoea at 1 year. However, no univariable association was found for the WHO Clinical Progression Scale, despite this scale being purposefully defined to characterise COVID-19 illness severity.^13^ In our exploratory analysis of post-COVID-19 dyspnoea at 1 year, a univariable association was found for patients with the most severe disease (WHO class 7–9: requiring intubation or mechanical ventilation), but this was not retained in the final model. Studies using PHOSP-COVID^7^^,^^9^ have found associations between having required invasive mechanical ventilation and patient-perceived recovery at 5 months and 1 year after discharge, but no associations with dyspnoea specifically.^9^ It is possible that length of hospital stay reflects duration of intubation or mechanical ventilation in participants with WHO class 7–9, and therefore provides a more nuanced picture of severity in patients with the most severe disease. In addition, long hospital stay could lead to deconditioning and frailty which might be part of the underlying mechanisms of post-COVID-19 dyspnoea. Further research is therefore needed to clarify the relationship between acute COVID-19 severity and post-COVID-19 dyspnoea, with a focus on treatment received and the negative consequences.

Unsurprisingly, most of the predictors we identified for improvements in dyspnoea between 5 months and 1 year showed inverse associations with long-term, post-COVID-19 dyspnoea, or worsening of dyspnoea over 1 year, with a few notable exceptions. Pre-existing respiratory disease and being an ex or current smoker were both associated with long-term, post-COVID-19 dyspnoea in patients hospitalised with COVID-19, or worsening of dyspnoea over 1 year in community controls, potentially reflecting accelerated decline in pulmonary function in these populations.^29^^,^^30^ Additionally, socioeconomic deprivation, as measured by the IMD 2019, was associated with increased risk of post-COVID-19 dyspnoea in hospitalised patients, which was consistent with a report from the Office for National Statistics with a representative sample.^3^ This association could be linked to air quality,^31^ which forms part of the IMD living environment domain.^32^ However, other studies have found little variation in patient-perceived recovery^9^ or risk of long COVID symptoms^33^ across IMD categories, and so further studies are needed to clarify associations between post-COVID-19 dyspnoea and socioeconomic deprivation.

This study has several strengths. We used data from two large, prospective, observational studies, both of which measured dyspnoea with the same widely used and validated scale. The two studies covered a full spectrum of hospitalised and non-hospitalised COVID-19 patients and uninfected participants, and particularly, by including a control population with follow-up measurements taken at the same intervals, we were able to explore whether the risk factors identified were specific to people with severe COVID-19. Both cohorts had multiple follow-up measurements that allowed us to examine the recovery trajectory of post-COVID-19 or all-cause dyspnoea, which is of clinical interest given the large number of affected patients and their unmet healthcare needs. Additionally, participants were recruited and followed up over similar time periods between cohorts and thus were more comparable.

This study also has several limitations. First, we assessed dyspnoea with the MRC Dyspnoea Scale, which is a measure of breathlessness outcome with responses framed in terms of how much breathlessness limits the respondent's activities.^12^ The MRC Dyspnoea Scale is therefore highly related to functionality, but does not quantify dyspnoea directly.^34^ Additionally, with five grades, it may not be sufficiently sensitive to change.^35^ However, it remains a widely used scale for both all-cause dyspnoea and post-COVID-19 dyspnoea, objectively captures the functional consequences and impacts of dyspnoea (prognostic of future healthcare need), and ensured our results can be easily compared with past and future studies. The different administration methods (in-person visit in PHOSP *vs* online survey in COVIDENCE UK) may have led to heterogeneity in dyspnoea measurement, but the consistent results between cohorts suggest no substantial influence on estimated associations. Second, the pre-COVID-19 dyspnoea scores from PHOSP-COVID participants were recalled at the 5-month visit, and thus may be subject to recall bias. However, this does not affect our primary analysis, which focused on measurements at 5-month and 1-year follow-up. Additionally, no COVIDENCE UK measurements were recalled, and comparison of exploratory analysis results with the community controls shows many predictors in common, lending strength to our analysis in PHOSP-COVID cohort. Third, our analysis of PHOSP-COVID participants was restricted to those able to attend both research visits, and thus may not include participants worst affected by post-COVID-19 dyspnoea. However, 13% of PHOSP-COVID participants graded their dyspnoea at the 5-month visit with a score of 5 ("too breathless to leave the house, or breathless when dressing/undressing"), showing that participants greatly affected by their dyspnoea were nonetheless represented. Fourth, as our sample size of community COVID-19 cases was limited, we only compared the risk factors for dyspnoea recovery between the cohort of hospitalised COVID-19 cases versus community-based non-COVID-19 participants in the main analysis. Therefore, our findings on post-COVID-19 dyspnoea are largely limited to people who had severe disease. However, evidence suggests that acute disease severity is a determinant of long COVID,^36^ which makes hospitalised patients a particular population of interest. We acknowledge that the use of a community-based control group is suboptimal, and future studies with patients hospitalised for a respiratory infection not due to SARS-Cov-2 could further strengthen the evidence. Finally, we had no data on whether participants with dyspnoea were being treated for their symptoms, and therefore were not able to explore the effect of treatment on outcomes.

Dyspnoea is a debilitating symptom that negatively impacts quality of life,^5^ and is a common long-term symptom among COVID-19 survivors.^3^ We show that patients hospitalised with COVID-19 who were older, female, obese, or with pre-existing anxiety or depression, or cardiovascular disease had reduced odds of improvement in long-term post-COVID-19 dyspnoea 1 year after discharge, highlighting a population at risk of enduring long COVID. Our finding that similar risk factors predicted persistent non-COVID-19 dyspnoea in the general population suggests that existing research on managing and treating dyspnoea may apply in COVID-19 survivors. With global weekly COVID-19 cases still in the millions,^1^ of whom up to 20% may be affected by long-term dyspnoea,^4^ intervention studies are urgently needed to identify treatment pathways for patients burdened by post-COVID-19 dyspnoea.

## Contributors

Aziz Sheikh and AM conceived this analysis and oversaw all aspects of the work. BZ and GV led on the analysis and the drafting of the manuscript. All authors contributed to data interpretation and critical review and revision of the manuscript. Aziz Sheikh and AM had full access to all the data in the study and had final responsibility for the decision to submit for publication.

## Data sharing statement

PHOSP-COVID: The protocol, consent form, definition and derivation of clinical characteristics and outcomes, training materials, regulatory documents, requests for data access and other relevant study materials are available online at https://www.phosp.org.

COVIDENCE UK: De-identified participant data will be made available upon reasonable request to the corresponding author.

## Declaration of interests

Aziz Sheikh received research grant to Institution from the National Institute of Health and Care Research (NIHR); participated in AstraZeneca's Thrombotic Thrombocytopenic TaskForce (unremunerated); served as an adviser to UK and Scottish Government COVID bodies (unremunerated). LGH received project grant funding to Institution from MedImmune, Novartis UK, Roche/Genentech and GlaxoSmithKline (GSK); payment for lectures from AstraZeneca, Novartis, Roche/Genentech, Sanofi, Circassia, GSK, Chiesi, Teva; travel funding from AstraZeneca, Chiesi, Novartis, Boehringer Ingelheim, Teva and GSK; attended advisory boards/lectures of Novartis, Roche/Genentech, GSK, Evelo Biosciences, Teva, Theravance and Vectura. ADS received grants unrelated to the current work from AstraZeneca, Bayer, GSK, Pfizer, Novartis; consulting fees from AstraZeneca, GSK, Insmed and Bayer; travel support from Chiesi, AstraZeneca and GSK; attended advisory board of Insmed. AH received funding from UK Research and Innovation, National Institute of Health Research, NIHR Manchester BRC; chair NIHR Translational Research Collaboration (unpaid). JDC received funding from AstraZeneca, Genentech, Gilead Sciences, GSK, Insmed, Grifols, Novartis, Boehringer Ingelheim; consulting fees from Astrazeneca, Chiesi, GSK, Insmed, Grifols, Novartis, Boehringer Ingelheim, Pfizer, Janssen, Antabio, Zambon. RAE received funding from NIHR, UKRI, Wolfson Foundation; consulting fees from AstraZeneca; payment for lecture from Boeringher; support for attending meetings from Chiesi; served as ERS Group 01.02 Pulmonary Rehabilitation Secretary (unpaid). AM received funding to Institution from Barts Charity, Pharma Nord, Fischer Family Foundation, DSM Nutritional Products, The Exilarch's Foundation, The Karl R Pfleger Foundation, The AIM Foundation, Synergy Biologics, Cytoplan Ltd, UK NIHR Clinical Research Network, Health Data Research UK BREATHE Hub, UK Research and Innovation Industrial Strategy Challenge Fund, Thornton & Ross, Warburtons, Matthew Isaacs (personal donation), Barbara Boucher (personal donation), Hyphens Pharma. Amisha Singapuri received funding to Institution from UKRI and NIHR. BR received funding from BHF Oxford CRE Transition Intermediate Clinical Research Fellowship; payment for lecture from Axcella Therapeutics. CEB received funding to Institution from MRC, NIHR, Leicester NIHR BRC, GSK, AstraZeneca, Sanofi, Regeneron, Roche, Genentech, BI, Novartis, Chiesi, 4Dpharma, Mologic; consulting fees to institution from GSK, AstraZeneca, Sanofi, Regeneron, Roche, Genentech, BI, Novartis, Chiesi, 4Dpharma, Mologic. MS received funding to Institution from UKRI-MRC and DHSC-NIHR. LVW received funding from UK Research and Innovation, GSK/Asthma + Lung UK, NIHR, Orion Pharma, GSK, Genentech, AstraZeneca; consulting fees to institution from Galapagos, Boehringer Ingelheim; travel support from Genentech; attended Advisory Board for Galapagos; served as Associate Editor for European Respiratory Journal. PEP received funding from NIHR. JKQ received funding to Institution from MRC, HDR UK, GSK, BI, asthma + lung UK, AstraZeneca; consulting fees from GSK, AstraZeneca, Insmed. MM received funding from the PHOSP Covid grant. Other authors declare no conflict of interests.
