# Supplementary material for: Determinants of recovery from post-COVID-19 dyspnoea: analysis of UK prospective cohorts of hospitalised COVID-19 patients and community-based controls
Source: Lancet Reg Health Eur. 2023 Apr 28;29:100635. doi: 10.1016/j.lanepe.2023.100635 (PMC10145209; doi:10.1016/j.lanepe.2023.100635)
Supplement: Supplementary appendix [file mmc1.docx]

**Determinants of recovery from post-COVID-19 dyspnoea: analysis of UK prospective cohorts of hospitalised COVID-19 patients and community-based controls**

Appendix

[STROBE checklist for observational studies 2](#_Toc129162178)

[*Figure S1:* Cohort profiles for the exploratory analysis on PHOSP-COVID (A) and COVIDENCE UK (B) 4](#_Toc129162179)

[*Table S1:* Sensitivity analyses for predicting improvement of dyspnoea symptoms from the 5-month to 1-year visits of PHOSP study 5](#_Toc129162180)

[*Table S2:* Sensitivity analyses for predicting improvement of dyspnoea symptoms from 5 to 12 months in COVIDENCE UK controls 6](#_Toc129162181)

[*Table S3:* Participant characteristics by recovery status of dyspnoea symptoms from 5 to 12 months after enrolment, for COVIDENCE UK COVID-19 cases 7](#_Toc129162182)

[*Table S4:* Associations between participant characteristics and improvement of dyspnoea symptoms from 5 to 12 months, for COVIDENCE UK cases and all eligible COVIDENCE UK participants, adjusted for COVID-19 case status 8](#_Toc129162183)

[*Table S5:* Participant characteristics by long-term dyspnoea status at 12 months 9](#_Toc129162184)

[PHOSP-COVID Study Collaborative Group 10](#_Toc129162185)

# **STROBE checklist for observational studies**

|  | | **Item No** | **Recommendation** | **Page  No** |
| --- | --- | --- | --- | --- |
| **Title and abstract** | | 1 | (*a*) Indicate the study’s design with a commonly used term in the title or the abstract | 1 |
|  |  |  | (*b*) Provide in the abstract an informative and balanced summary of what was done and what was found | 4 |
| **Introduction** | | | | |
| Background/rationale | | 2 | Explain the scientific background and rationale for the investigation being reported | 6 |
| Objectives | | 3 | State specific objectives, including any prespecified hypotheses | 7 |
| **Methods** | | | | |
| Study design | | 4 | Present key elements of study design early in the paper | 7 |
| Setting | | 5 | Describe the setting, locations, and relevant dates, including periods of recruitment, exposure, follow-up, and data collection | 7–8 |
| Participants | | 6 | (*a*) *Cohort study*—Give the eligibility criteria, and the sources and methods of selection of participants. Describe methods of follow-up  *Case-control study*—Give the eligibility criteria, and the sources and methods of case ascertainment and control selection. Give the rationale for the choice of cases and controls  *Cross-sectional study*—Give the eligibility criteria, and the sources and methods of selection of participants | 7–9 |
|  |  |  | (*b*) *Cohort study*—For matched studies, give matching criteria and number of exposed and unexposed  *Case-control study*—For matched studies, give matching criteria and the number of controls per case | NA |
| Variables | | 7 | Clearly define all outcomes, exposures, predictors, potential confounders, and effect modifiers. Give diagnostic criteria, if applicable | 8–9 |
| Data sources/ measurement | | 8 | For each variable of interest, give sources of data and details of methods of assessment (measurement). Describe comparability of assessment methods if there is more than one group | 7–9 |
| Bias | | 9 | Describe any efforts to address potential sources of bias | 8-10 |
| Study size | | 10 | Explain how the study size was arrived at | Fig 1 |
| Quantitative variables | | 11 | Explain how quantitative variables were handled in the analyses. If applicable, describe which groupings were chosen and why | 8–10 |
| Statistical methods | | 12 | (*a*) Describe all statistical methods, including those used to control for confounding | 9–10 |
|  |  |  | (*b*) Describe any methods used to examine subgroups and interactions | 10 |
|  |  |  | (*c*) Explain how missing data were addressed | 10 |
|  |  |  | (*d*) *Cohort study*—If applicable, explain how loss to follow-up was addressed  *Case-control study*—If applicable, explain how matching of cases and controls was addressed  *Cross-sectional study*—If applicable, describe analytical methods taking account of sampling strategy | 7-8 |
|  |  |  | (*e*) Describe any sensitivity analyses | 9-10 |
| **Results** | | | | |
| Participants | 13 | (a) Report numbers of individuals at each stage of study—eg numbers potentially eligible, examined for eligibility, confirmed eligible, included in the study, completing follow-up, and analysed | | 11, Fig 1 |
|  |  | (b) Give reasons for non-participation at each stage | | 11, Fig 1 |
|  |  | (c) Consider use of a flow diagram | | Fig 1 |
| Descriptive data | 14 | (a) Give characteristics of study participants (eg demographic, clinical, social) and information on exposures and potential confounders | | 11-12 |
|  |  | (b) Indicate number of participants with missing data for each variable of interest | | Table 1 |
|  |  | (c) *Cohort study*—Summarise follow-up time (eg, average and total amount) | | NA |
| Outcome data | 15 | *Cohort study*—Report numbers of outcome events or summary measures over time | | 11-13 |
|  |  | *Case-control study—*Report numbers in each exposure category, or summary measures of exposure | | ·· |
|  |  | *Cross-sectional study—*Report numbers of outcome events or summary measures | | ·· |
| Main results | 16 | (*a*) Give unadjusted estimates and, if applicable, confounder-adjusted estimates and their precision (eg, 95% confidence interval). Make clear which confounders were adjusted for and why they were included | | Tables 2-3 |
|  |  | (*b*) Report category boundaries when continuous variables were categorized | | Tables 2-3 |
|  |  | (*c*) If relevant, consider translating estimates of relative risk into absolute risk for a meaningful time period | | NA |
| Other analyses | 17 | Report other analyses done—eg analyses of subgroups and interactions, and sensitivity analyses | | 11-13, appendix tables |
| **Discussion** | | | | |
| Key results | 18 | Summarise key results with reference to study objectives | | 13–14 |
| Limitations | 19 | Discuss limitations of the study, taking into account sources of potential bias or imprecision. Discuss both direction and magnitude of any potential bias | | 16-17 |
| Interpretation | 20 | Give a cautious overall interpretation of results considering objectives, limitations, multiplicity of analyses, results from similar studies, and other relevant evidence | | 13–17 |
| Generalisability | 21 | Discuss the generalisability (external validity) of the study results | | 16-17 |
| **Other information** | | | | |
| Funding | 22 | Give the source of funding and the role of the funders for the present study and, if applicable, for the original study on which the present article is based | | 18 |

# ***Figure S1:* Cohort profiles for the exploratory analysis on PHOSP-COVID (A) and COVIDENCE UK (B)**


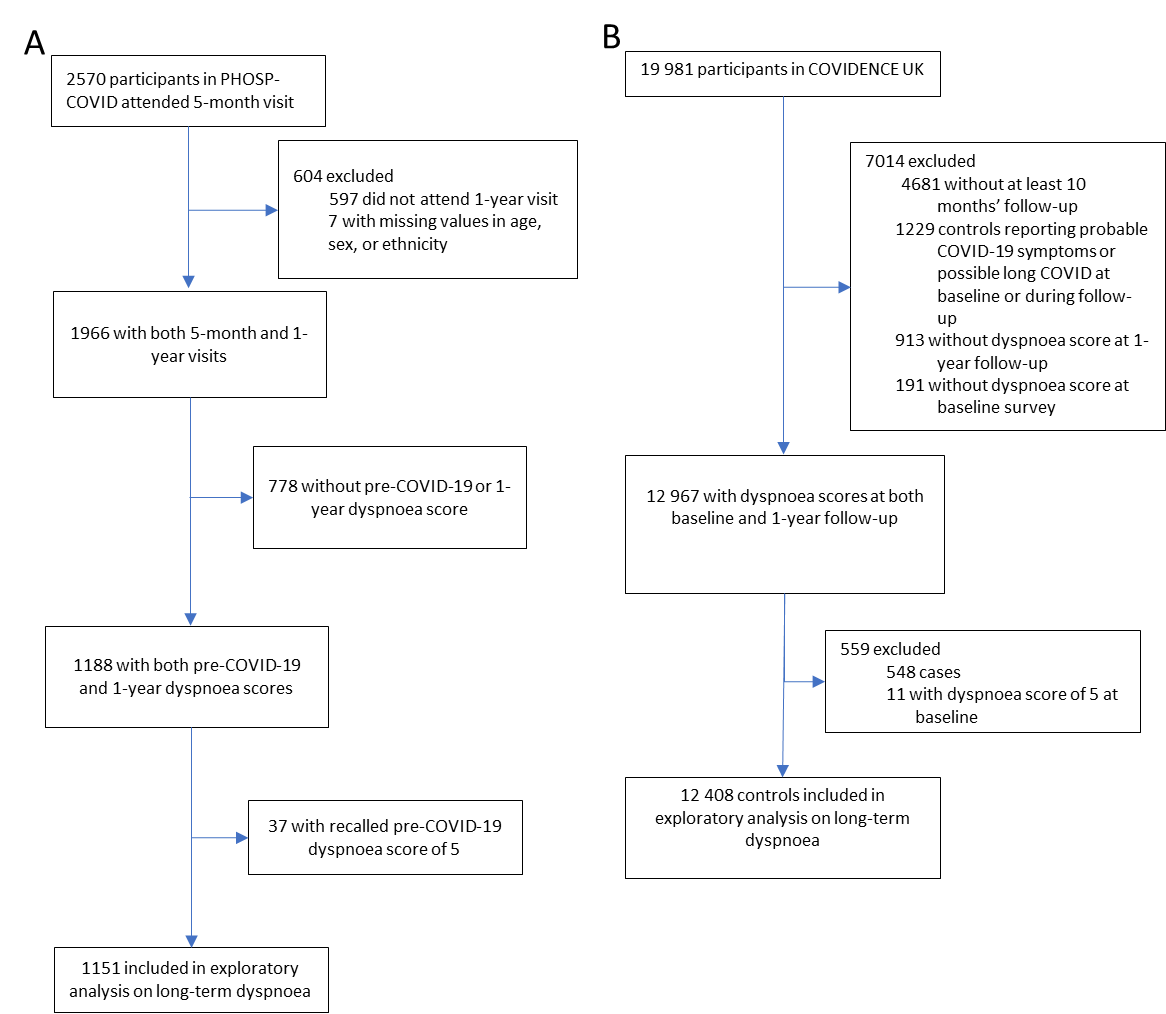


|  | **Fully adjusted model (n=990)** | **Complete-case analysis (n=692)** | **Multiple imputation (n=990)** | **Excluding participants with improved dyspnoea at 5 months  (MRC_pre_ ≥ MRC_5m_; n=797)** | **Adjusted for calendar date and visit interval (n=984)** |
| --- | --- | --- | --- | --- | --- |
| Age, per year | 0.98 (0.97–0.99) | 0.98 (0.97–1.00) | 0.98 (0.97–0.99) | 0.98 (0.97–1.00) | 0.98 (0.97–0.99) |
| Sex |  |  |  |  |  |
| Female | 0.63 (0.47–0.85) | 0.62 (0.44–0.88) | 0.65 (0.49–0.86) | 0.65 (0.47–0.88) | 0.63 (0.48–0.84) |
| Male | 1 (ref) | 1 (ref) | 1 (ref) | 1 (ref) | 1 (ref) |
| Ethnicity |  |  |  |  |  |
| White | 1 (ref) | ·· | ·· | ·· | ·· |
| Black | 1.23 (0.71–2.11) | ·· | ·· | ·· | ·· |
| South Asian | 0.94 (0.58–1.52) | ·· | ·· | ·· | ·· |
| Mixed | 0.96 (0.39–2.36) | ·· | ·· | ·· | ·· |
| Other | 0.94 (0.45–1.94) | ·· | ·· | ·· | ·· |
| IMD quintile |  |  |  |  |  |
| 1 (least deprived) | 1 (ref) | ·· | ·· | ·· | ·· |
| 2 | 0.84 (0.53–1.34) | ·· | ·· | ·· | ·· |
| 3 | 0.72 (0.45–1.13) | ·· | ·· | ·· | ·· |
| 4 | 0.71 (0.46–1.09) | ·· | ·· | ·· | ·· |
| 5 (most deprived) | 0.74 (0.47–1.14) | ·· | ·· | ·· | ·· |
| BMI, kg/m² |  |  |  |  |  |
| <25 (normal or underweight) | 1 (ref) | 1 (ref) | 1 (ref) | 1 (ref) | 1 (ref) |
| 25 to <30 (overweight) | 0.68 (0.38–1.22) | 0.67 (0.38–1.17) | 0.66 (0.33–1.34) | 0.62 (0.34–1.16) | 0.69 (0.39–1.21) |
| 30 to <40 (obese) | 0.54 (0.31–0.95) | 0.55 (0.32–0.95) | 0.59 (0.33–1.07) | 0.51 (0.28–0.92) | 0.53 (0.31–0.92) |
| ≥40 (severely obese) | 0.35 (0.17–0.72) | 0.35 (0.17–0.70) | 0.37 (0.14–0.93) | 0.28 (0.13–0.60) | 0.34 (0.17–0.69) |
| Current or ex-smoker | 0.80 (0.60–1.08) | ·· | ·· | ·· | ·· |
| Comorbidity |  |  |  |  |  |
| Cardiovascular disease | 0.77 (0.57–1.04) | 0.70 (0.49–1.00) | 0.74 (0.55–0.99) | 0.81 (0.59–1.11) | 0.74 (0.55–1.00) |
| Respiratory disease | 1.02 (0.75–1.39) | ·· | ·· | ·· | ·· |
| Depression or anxiety | 0.66 (0.46–0.95) | 0.61 (0.40–0.94) | 0.66 (0.46–0.94) | 0.54 (0.36–0.81) | 0.66 (0.46–0.95) |
| Length of hospital stay, per day | 0.99 (0.98–1.00) | 0.99 (0.98–1.00) | 0.99 (0.98–1.00) | 0.99 (0.98–0.99) | 0.99 (0.98–1.00) |
| WHO clinical progression scale |  |  |  |  |  |
| WHO – class 3–4 | 1 (ref) | ·· | ·· | ·· | ·· |
| WHO – class 5 | 1.22 (0.78–1.92) | ·· | ·· | ·· | ·· |
| WHO – class 6 | 1.49 (0.88–2.51) | ·· | ·· | ·· | ·· |
| WHO – class 7–9 | 1.02 (0.52–1.98) | ·· | ·· | ·· | ·· |
| Pulmonary embolism during hospitalisation | 1.40 (0.86–2.29) | ·· | ·· | ·· | ·· |
| Treatment during hospitalisation |  |  |  |  |  |
| Proning | 1.07 (0.70–1.64) | ·· | ·· | ·· | ·· |
| Antibiotic therapy | 0.85 (0.60–1.21) | ·· | ·· | ·· | ·· |
| Systemic (oral or IV) steroids | 1.11 (0.80–1.53) | ·· | ·· | ·· | ·· |
| Therapeutic dose anticoagulation | 1.07 (0.78–1.47) | ·· | ·· | ·· | ·· |
| MRC Dyspnoea score at 5 months * |  |  |  |  |  |
| 2 | 1 (ref) | 1 (ref) | 1 (ref) | 1 (ref) | 1 (ref) |
| 3 | 1.36 (0.96–1.93) | 1.49 (1.00–2.22) | 1.35 (0.96–1.90) | 1.28 (0.88–1.86) | 1.30 (0.92–1.82) |
| 4 | 1.88 (1.26–2.81) | 2.04 (1.28–3.26) | 1.82 (1.22–2.71) | 1.80 (1.18–2.76) | 1.73 (1.17–2.55) |
| 5 | 2.66 (1.68–4.21) | 3.07 (1.78–5.31) | 2.69 (1.71–4.24) | 2.59 (1.60–4.20) | 2.55 (1.62–3.99) |

Data are odds ratio (95% CI). No significant non-linear effect was detected for age or duration of hospital stay. BMI=body-mass index. IMD=Index of Multiple Deprivation. IV=intravenous. MRC=Medical Research Council. WHO=World Health Organization. *Participants with no dyspnoea symptoms (MRC score = 1) at the 5-month visit were excluded from these analyses.

# ***Table S1:* Sensitivity analyses for predicting improvement of dyspnoea symptoms from the 5-month to 1-year visits of PHOSP study**

|  | **Fully adjusted model (n=3309)** | **Complete-case analysis (n=3300)** | **Multiple imputation (n=3309)** | **Excluding participants with improved dyspnoea at 5 months  (MRC_0_ ≥ MRC_5_; n=1178)** | **Adjusted for calendar date (n=3309)** |
| --- | --- | --- | --- | --- | --- |
| Age, per year | 0.99 (0.99–1.00) | ·· | ·· | ·· | ·· |
| Sex |  |  |  |  |  |
| Female | 0.72 (0.60–0.86) | 0.74 (0.62–0.89) | 0.74 (0.61–0.88) | 0.75 (0.57–0.99) | 0.75 (0.62–0.90) |
| Male | 1 (ref) | 1 (ref) | 1 (ref) | 1 (ref) | 1 (ref) |
| Ethnicity |  |  |  |  |  |
| White | 1 (ref) | ·· | ·· | ·· | ·· |
| Black | 1.92 (0.67–5.51) | ·· | ·· | ·· | ·· |
| South Asian | 0.57 (0.25–1.31) | ·· | ·· | ·· | ·· |
| Mixed | 0.96 (0.51–1.80) | ·· | ·· | ·· | ·· |
| Other | 1.05 (0.51–2.19) | ·· | ·· | ·· | ·· |
| IMD quintile |  |  |  |  |  |
| 1 (least deprived) | 1 (ref) | ·· | ·· | ·· | ·· |
| 2 | 0.93 (0.76–1.14) | ·· | ·· | ·· | ·· |
| 3 | 0.83 (0.66–1.03) | ·· | ·· | ·· | ·· |
| 4 | 0.80 (0.62–1.03) | ·· | ·· | ·· | ·· |
| 5 (most deprived) | 0.70 (0.51–0.97) | ·· | ·· | ·· | ·· |
| BMI, kg/m² |  |  |  |  |  |
| <25 (normal or underweight) | 1 (ref) | 1 (ref) | 1 (ref) | 1 (ref) | 1 (ref) |
| 25 to <30 (overweight) | 0.90 (0.75–1.09) | 0.89 (0.74–1.08) | 0.89 (0.74–1.08) | 0.93 (0.70–1.23) | 0.89 (0.74–1.08) |
| 30 to <40 (obese) | 0.62 (0.51–0.76) | 0.62 (0.50–0.75) | 0.61 (0.50–0.75) | 0.69 (0.51–0.94) | 0.62 (0.50–0.76) |
| ≥40 (severely obese) | 0.50 (0.34–0.74) | 0.51 (0.34–0.75) | 0.51 (0.34–0.75) | 0.46 (0.24–0.89) | 0.51 (0.34–0.75) |
| Current or ex-smoker | 1.00 (0.85–1.17) | ·· | ·· | ·· | ·· |
| Comorbidity |  |  |  |  |  |
| Cardiovascular disease | 0.60 (0.42–0.86) | 0.57 (0.40–0.81) | 0.57 (0.40–0.81) | 0.91 (0.52–1.60) | 0.57 (0.40–0.81) |
| Respiratory disease | 0.88 (0.73–1.06) | ·· | ·· | ·· | ·· |
| Depression or anxiety | 0.82 (0.69–0.97) | 0.83 (0.71–0.98) | 0.83 (0.70–0.98) | 0.80 (0.62–1.03) | 0.84 (0.71–0.99) |
| MRC Dyspnoea score at 5 months * |  |  |  |  |  |
| 2 | 1 (ref) | 1 (ref) | 1 (ref) | 1 (ref) | 1 (ref) |
| 3 | 2.15 (1.68–2.75) | 2.09 (1.64–2.68) | 2.08 (1.63–2.66) | 1.67 (1.18–2.36) | 2.10 (1.65–2.69) |
| 4 | 4.10 (2.55–6.57) | 3.79 (2.37–6.05) | 3.61 (2.28–5.74) | 4.19 (2.12–8.28) | 3.86 (2.42–6.17) |
| 5 | 3.74 (0.74–19.00) | 3.37 (0.66–17.08) | 3.37 (0.67–17.11) | Not estimable | 3.49 (0.69–17.64) |

Data are odds ratio (95% CI). Inclusion of a square term for age was not assessed in the final model, as age was not a significant predictor. BMI=body-mass index. IMD=Index of Multiple Deprivation. IV=intravenous. MRC=Medical Research Council. WHO=World Health Organization. *Participants with no dyspnoea symptoms (MRC score = 1) at the 5-month visit were excluded from these analyses.

# ***Table S2:* Sensitivity analyses for predicting improvement of dyspnoea symptoms from 5 to 12 months in COVIDENCE UK controls**

|  | **All participants (n=303)** | **Improved dyspnoea (n=92)** | **Worse or persistent dyspnoea (n=211)** |
| --- | --- | --- | --- |
| Sociodemographics | | | |
| Age, years | 55.3 (11.7) | 53.7 (11.5) | 55.9 (11.7) |
| Sex |  |  |  |
| Female | 231 (76.2%) | 63 (68.5%) | 168 (79.6%) |
| Male | 72 (23.8%) | 29 (31.5%) | 43 (20.4%) |
| Ethnicity |  |  |  |
| White | 287 (94.7%) | 91 (98.9%) | 196 (92.9%) |
| Black | 2 (0.7%) | 0 | 2 (0.9%) |
| South Asian | 6 (2.0%) | 1 (1.1%) | 5 (2.4%) |
| Mixed | 6 (2.0%) | 0 | 6 (2.8%) |
| Other | 2 (0.7%) | 0 | 2 (0.9%) |
| IMD quintile |  |  |  |
| 1 (least deprived) | 94 (31.0%) | 30 (32.6%) | 64 (30.3%) |
| 2 | 75 (24.8%) | 26 (28.3%) | 49 (23.2%) |
| 3 | 53 (17.5%) | 20 (21.7%) | 33 (15.6%) |
| 4 | 49 (16.2%) | 9 (9.8%) | 40 (19.0%) |
| 5 (most deprived) | 32 (10.6%) | 7 (7.6%) | 25 (11.8%) |
| Clinical characteristics | | | |
| BMI, kg/m² | 28.8 (6.3) | 27.6 (4.6) | 29.3 (6.8) |
| <25 (normal or underweight) | 92/302 (30.5%) | 28 (30.4%) | 64/210 (30.5%) |
| 25 to <30 (overweight) | 101/302 (33.4%) | 38 (41.3%) | 63/210 (30.0%) |
| 30 to <40 (obese) | 90/302 (29.8%) | 25 (27.2%) | 65/210 (31.0%) |
| ≥40 (severely obese) | 19/302 (6.3%) | 1 (1.1%) | 18/210 (8.6%) |
| Current or ex-smoker | 142 (46.9%) | 36 (39.1%) | 106 (50.2%) |
| Comorbidity |  |  |  |
| Cardiovascular disease | 18 (5.9%) | 4 (4.3%) | 14 (6.6%) |
| Respiratory disease | 63 (20.8%) | 16 (17.4%) | 47 (22.3%) |
| Depression or anxiety | 129 (42.6%) | 36 (39.1%) | 93 (44.1%) |
| MRC Dyspnoea score at 5 months* |  |  |  |
| 2 | 230 (75.9%) | 58 (63.0%) | 172 (81.5%) |
| 3 | 54 (17.8%) | 23 (25.0%) | 31 (14.7%) |
| 4 | 16 (5.3%) | 8 (8.7%) | 8 (3.8%) |
| 5 | 3 (1.0%) | 3 (3.3%) | 0 |
| Hospitalisation characteristics | | | |
| Hospitalised with COVID-19 | 66 (21.8%) | 22 (23.9%) | 44 (20.9%) |
| Length of hospital stay, days† | 2 (0–6) | 2 (0–5) | 2 (0–7) |

Data are n (%), mean (SD), or median (IQR). BMI=body-mass index. IMD=Index of Multiple Deprivation. MRC=Medical Research Council. *Participants with no dyspnoea symptoms (MRC score = 1) at 5 months were excluded from this analysis. †Length of stay missing for two participants with improved dyspnoea and three participants with worse or persistent dyspnoea.

# ***Table S3:* Participant characteristics by recovery status of dyspnoea symptoms from 5 to 12 months after enrolment, for COVIDENCE UK COVID-19 cases**

|  | **COVIDENCE UK cases (n=303)** | | **COVIDENCE UK interaction model (n=3612)** | |
| --- | --- | --- | --- | --- |
|  | Univariable | Multivariable | Univariable | Multivariable |
| Age, per year | 0.98 (0.96–1.00) | 0.97 (0.95–0.99) | 1.00 (0.99–1.00) | 0.99 (0.99–1.00) |
| Sex |  |  |  |  |
| Female | 0.56 (0.32–0.97) | 0.44 (0.23–0.83) | 0.73 (0.62–0.86) | 0.70 (0.58–0.83) |
| Male | 1 (ref) | 1 (ref) | 1 (ref) | 1 (ref) |
| Ethnicity |  |  |  |  |
| White | 1 (ref) | ·· | 1 (ref) | ·· |
| Black | Not estimable | ·· | 1.40 (0.52–3.80) | ·· |
| South Asian | 0.43 (0.05–3.74) | ·· | 0.57 (0.26–1.23) | ·· |
| Mixed | Not estimable | ·· | 0.78 (0.43–1.43) | ·· |
| Other | Not estimable | ·· | 1.09 (0.53–2.21) | ·· |
| IMD quintile |  |  |  |  |
| 1 (least deprived) | 1 (ref) | ·· | 1 (ref) | 1 (ref) |
| 2 | 1.13 (0.59–2.15) | ·· | 0.95 (0.79–1.16) | 0.94 (0.77–1.15) |
| 3 | 1.29 (0.64–2.62) | ·· | 0.87 (0.71–1.07) | 0.86 (0.69–1.06) |
| 4 | 0.48 (0.21–1.12) | ·· | 0.76 (0.61–0.96) | 0.77 (0.61–0.98) |
| 5 (most deprived) | 0.60 (0.23–1.53) | ·· | 0.72 (0.53–0.96) | 0.68 (0.50–0.93) |
| BMI, kg/m² |  |  |  |  |
| <25 (normal or underweight) | 1 (ref) | 1 (ref) | 1 (ref) | 1 (ref) |
| 25 to <30 (overweight) | 1.38 (0.76–2.51) | 1.87 (0.95–3.66) | 0.96 (0.80–1.14) | 0.95 (0.79–1.13) |
| 30 to <40 (obese) | 0.88 (0.46–1.67) | 0.88 (0.44–1.75) | 0.67 (0.56–0.81) | 0.64 (0.53–0.78) |
| ≥40 (severely obese) | 0.13 (0.02–1.00) | Not estimable | 0.63 (0.44–0.91) | 0.51 (0.34–0.75) |
| Current or ex-smoker | 0.64 (0.39–1.05) | ·· | 0.93 (0.80–1.07) | ·· |
| Comorbidity |  |  |  |  |
| Cardiovascular disease | 0.64 (0.20–2.00) | ·· | 0.73 (0.53–1.01) | 0.60 (0.42–0.84) |
| Respiratory disease | 0.73 (0.39–1.38) | 0.47 (0.22–1.00) | 0.92 (0.77–1.09) | 0.84 (0.70–1.00) |
| Depression or anxiety | 0.82 (0.50–1.34) | ·· | 0.87 (0.75–1.01) | 0.82 (0.70–0.96) |
| MRC Dyspnoea score at 5 months * |  |  |  |  |
| 2 | 1 (ref) | 1 (ref) | 1 (ref) | 1 (ref) |
| 3 | 2.20 (1.19–4.07) | 2.76 (1.38–5.54) | 1.83 (1.47–2.27) | 2.22 (1.76–2.79) |
| 4 | 2.97 (1.06–8.26) | 3.93 (1.22–12.66) | 2.83 (1.89–4.25) | 4.00 (2.59–6.17) |
| 5 | Not estimable | Not estimable | 5.78 (1.44–23.15) | 7.24 (1.76–29.76) |
| COVID-19 case status |  |  |  |  |
| Case | ·· | ·· | 1.14 (0.88–1.47) | 0.97 (0.74–1.27) |
| Control | ·· | ·· | 1 (ref) | 1 (ref) |

Data are odds ratio (95% CI). For the interaction model, any significant predictors retained with backwards selection in the multivariable model were then interacted with COVID-19 case status, and then run through backwards selection again. No significant interactions were retained in the final model. BMI=body-mass index. IMD=Index of Multiple Deprivation. MRC=Medical Research Council. *Participants with no dyspnoea symptoms (MRC score = 1) at 5 months were excluded from this analysis.

# ***Table S4:* Associations between participant characteristics and improvement of dyspnoea symptoms from 5 to 12 months, for COVIDENCE UK cases and all eligible COVIDENCE UK participants, adjusted for COVID-19 case status**

|  | **PHOSP (hospitalised COVID-19 cases)** | | | | **COVIDENCE UK (community controls)** | | |
| --- | --- | --- | --- | --- | --- | --- | --- |
|  | All participants (n=1151) | Long-term worsening of dyspnoea (n=609) | Stable or improved dyspnoea (n=542) | All participants (n=12 408) | | Onset or worsening of dyspnoea (n=1324) | Stable or improved dyspnoea (n=11 084) |
| **Sociodemographics** | | | | | | | |
| Age, years | 59.3 (12.0) | 59.3 (11.5) | 59.4 (12.6) | 61.0 (12.4) | | 60.7 (13.6) | 61.0 (12.2) |
| Sex |  |  |  |  | |  |  |
| Female | 439 (38.1%) | 268 (44.0%) | 171 (31.6%) | 8689 (70.0%) | | 977 (73.8%) | 7712 (69.6%) |
| Male | 712 (61.9%) | 341 (56.0%) | 371 (68.5%) | 3719 (30.0%) | | 347 (26.2%) | 3372 (30.4%) |
| Ethnicity |  |  |  |  | |  |  |
| White | 884 (76.8%) | 464 (76.2%) | 420 (77.5%) | 11 887 (95.8%) | | 1261 (95.2%) | 10 626 (95.9%) |
| Black | 79 (6.9%) | 46 (7.6%) | 33 (6.1%) | 60 (0.5%) | | 7 (0.5%) | 53 (0.5%) |
| South Asian | 116 (10.1%) | 59 (9.7%) | 57 (10.5%) | 172 (1.4%) | | 21 (1.6%) | 151 (1.4%) |
| Mixed | 26 (2.3%) | 11 (1.8%) | 15 (2.8%) | 159 (1.3%) | | 25 (1.9%) | 134 (1.2%) |
| Other | 46 (4.0%) | 29 (4.8%) | 17 (3.1%) | 130 (1.0%) | | 10 (0.8%) | 120 (1.1%) |
| IMD quintile |  |  |  |  | |  |  |
| 1 (least deprived) | 229/1147 (20.0%) | 102/608 (16.8%) | 127/539 (23.6%) | 4059 (32.7%) | | 409 (30.9%) | 3650 (32.9%) |
| 2 | 209/1147 (18.2%) | 103/608 (16.9%) | 106/539 (19.7%) | 3341 (26.9%) | | 349 (26.4%) | 2992 (27.0%) |
| 3 | 208/1147 (18.1%) | 116/608 (19.1%) | 92/539 (17.1%) | 2569 (20.7%) | | 279 (21.1%) | 2290 (20.7%) |
| 4 | 259/1147 (22.6%) | 143/608 (23.5%) | 116/539 (21.5%) | 1663 (13.4%) | | 190 (14.4%) | 1473 (13.3%) |
| 5 (most deprived) | 242/1147 (21.1%) | 144/608 (23.7%) | 98/539 (18.2%) | 776 (6.3%) | | 97 (7.3%) | 679 (6.1%) |
| **Clinical characteristics** | | | | | | | |
| BMI, kg/m² | 31.9 (7.0) | 32.7 (7.2) | 31.0 (6.7) | 26.1 (5.2) | | 27.8 (6.0) | 25.9 (5.0) |
| <25 (normal or underweight) | 97/772 (12.6%) | 39/416 (9.4%) | 58/356 (16.3%) | 6176 (49.8%) | | 488 (36.9%) | 5688 (51.3%) |
| 25 to <30 (overweight) | 235/772 (30.4%) | 116/416 (27.9%) | 119/356 (33.4%) | 3960 (31.9%) | | 446 (33.7%) | 3514 (31.7%) |
| 30 to <40 (obese) | 356/772 (46.1%) | 204/416 (49.0%) | 152/356 (42.7%) | 2014 (16.2%) | | 337 (25.5%) | 1677 (15.1%) |
| ≥40 (severely obese) | 84/772 (10.9%) | 57/416 (13.7%) | 27/356 (7.6%) | 258 (2.1%) | | 53 (4.0%) | 205 (1.8%) |
| Current or ex-smoker | 458/1057 (43.3%) | 264/561 (47.1%) | 194/496 (39.1%) | 5303 (42.7%) | | 653 (49.3%) | 4650 (42.0%) |
| Comorbidity |  |  |  |  | |  |  |
| Cardiovascular disease | 544 (47.3%) | 291 (47.8%) | 253 (46.7%) | 473 (3.8%) | | 64 (4.8%) | 409 (3.7%) |
| Respiratory disease | 290 (25.2%) | 141 (23.2%) | 149 (27.5%) | 2140 (17.2%) | | 265 (20.0%) | 1875 (16.9%) |
| Depression or anxiety | 173 (15.0%) | 102 (16.8%) | 71 (13.1%) | 3028 (24.4%) | | 425 (32.1%) | 2603 (23.5%) |
| MRC Dyspnoea score before COVID-19 or at baseline* |  |  |  |  | |  |  |
| 1 | 721 (62.6%) | 430 (70.6%) | 291 (53.7%) | 9059 (73.0%) | | 1131 (85.4%) | 7928 (71.5%) |
| 2 | 211 (18.3%) | 104 (17.1%) | 107 (19.7%) | 2975 (24.0%) | | 169 (12.8%) | 2806 (25.3%) |
| 3 | 148 (12.9%) | 52 (8.5%) | 96 (17.7%) | 295 (2.4%) | | 21 (1.6%) | 274 (2.5%) |
| 4 | 71 (6.2%) | 23 (3.8%) | 48 (8.9%) | 79 (0.6%) | | 3 (0.2%) | 76 (0.7%) |
| **Hospitalisation characteristics** | | | | | | | |
| Length of hospital stay, days | 8 (4–16) | 9 (5–20) | 7 (4–12) | ·· | | ·· | ·· |
| WHO clinical progression scale |  |  |  |  | |  |  |
| WHO – class 3–4 | 167 (14.5%) | 88 (14.5%) | 79 (14.6%) | ·· | | ·· | ·· |
| WHO – class 5 | 513 (44.6%) | 244 (40.1%) | 269 (49.6%) | ·· | | ·· | ·· |
| WHO – class 6 | 257 (22.3%) | 134 (22.0%) | 123 (22.7%) | ·· | | ·· | ·· |
| WHO – class 7–9 | 214 (18.6%) | 143 (23.5%) | 71 (13.1%) | ·· | | ·· | ·· |
| Pulmonary embolism during hospitalisation | 113/1098 (10.3%) | 63/578 (10.9%) | 50/520 (9.6%) | ·· | | ·· | ·· |
| Treatment during hospitalisation |  |  |  |  | |  |  |
| Proning | 221/1028 (21.5%) | 126/533 (23.6%) | 95/495 (19.2%) | ·· | | ·· | ·· |
| Antibiotic therapy | 879/1115 (78.8%) | 472/592 (79.7%) | 407/523 (77.8%) | ·· | | ·· | ·· |
| Systemic (oral or IV) steroids | 634/1090 (58.2%) | 330/574 (57.5%) | 304/516 (58.9%) | ·· | | ·· | ·· |
| Therapeutic dose anticoagulation | 504/1086 (46.4%) | 266/571 (46.6%) | 238/515 (46.2%) | ·· | | ·· | ·· |

Note: SD=standard deviation; BMI=body mass index; IQR=interquartile range; WHO=World Health Organization; IV=intravenous; MRC=Medical Research Council. * Patients who had the highest degree of dyspnoea (score=5) before COVID-19 were excluded from this analysis.

# ***Table S5:* Participant characteristics by long-term dyspnoea status at 12 months**

# **PHOSP-COVID Study Collaborative Group**

Core Management Group

Chief Investigator C E Brightling, Members R A Evans (Lead Co-I), L V Wain (Lead Co-I), J D Chalmers, V C Harris, L P Ho, A Horsley, M Marks, K Poinasamy, B Raman, A Shikotra, A Singapuri

PHOSP-COVID Study Central Coordinating Team

C E Brightling (Chief Investigator), R A Evans (Lead Co-I), L V Wain (Lead Co-I), R Dowling, C Edwardson, O Elneima, S Finney, N J Greening, B Hargadon, V C Harris, L Houchen--Wolloff, O C Leavy, H J C McAuley, C Overton, T Plekhanova, R M Saunders, M Sereno, A Singapuri, A Shikotra, C Taylor, S Terry, C Tong, B Zhao

Steering Committee

Co-chairs D Lomas, E Sapey, Institution representatives C Berry, C E Bolton, N Brunskill, E R Chilvers, R Djukanovic, Y Ellis, D Forton, N French, J George, N A Hanley, N Hart, L McGarvey, N Maskell, H McShane, M Parkes, D Peckham, P Pfeffer, A Sayer, A Sheikh, A A R Thompson, N Williams and core management group representation

Executive Board

Chair C E Brightling, representation from the core management group, each working group and platforms

Platforms

Bioresource

W Greenhalf (Co-Lead), M G Semple (Co-Lead), M Ashworth, H E Hardwick, L Lavelle-Langham, W Reynolds, M Sereno, R M Saunders, A Singapuri, V Shaw, A Shikotra, B Venson, L V Wain

Data Hub

A B Docherty (Co-Lead), E M Harrison (Co-Lead), A Sheikh (Co-Lead), J K Baillie, C E Brightling, L Daines, R Free, R A Evans, S Kerr, O C Leavy, N I Lone, H J C McAuley, R Pius, J Quint, M Richardson, M Sereno, M Thorpe, L V Wain

Imaging Alliance

M Halling-Brown (Co-Lead), F Gleeson (Co-Lead), J Jacob (Co-Lead), S Neubauer (Co-Lead) B Raman (Co-Lead) S Siddiqui (Co-Lead) J M Wild (Co-Lead), S Aslani, P Jezzard, H Lamlum, W Lilaonitkul, E Tunnicliffe, J Willoughby

Omics

L V Wain (Co-Lead), J K Baillie (Co-Lead), H Baxendale, C E Brightling, M Brown, J D Chalmers, R A Evans, B Gooptu, W Greenhalf, H E Hardwick, R G Jenkins, D Jones, I Koychev, C Langenberg, A Lawrie, P L Molyneaux, A Shikotra, J Pearl, M Ralser, N Sattar, R M Saunders, J T Scott, T Shaw, D Thomas, D Wilkinson

Working Groups

Airways

L G Heaney (Co-Lead), A De Soyza (Co-Lead), D Adeloye, C E Brightling, J S Brown, J Busby, J D Chalmers, C Echevarria, L Daines, O Elneima, RA Evans, J R Hurst, P Novotny, P Pfeffer, K Poinasamy, J Quint, I Rudan, E Sapey, M Shankar-Hari, A Sheikh, S Siddiqui, S Walker, B Zheng

Brain

J R Geddes (Lead), M Hotopf (Co-Lead), K Abel, R Ahmed, L Allan, C Armour, D Baguley, D Baldwin, C Ballard, K Bhui, G Breen, M Broome, T Brugha, E Bullmore, D Burn, F Callard, J Cavanagh, T Chalder, D Clark, A David, B Deakin, H Dobson, B Elliott, J Evans, R Francis, E Guthrie, P Harrison, M Henderson, A Hosseini, N Huneke, M Husain, T Jackson, I Jones, T Kabir, P Kitterick, A Korszun, I Koychev, J Kwan, A Lingford-Hughes, P Mansoori, H McAllister-Williams, K McIvor, L Milligan, R Morriss, E Mukaetova-Ladinska, K Munro, A Nevado-Holgado, T Nicholson, S Paddick, C Pariante, J Pimm, K Saunders, M Sharpe, G Simons, R Upthegrove, S Wessely

Cardiac

G P McCann (Lead), S Amoils, C Antoniades, A Banerjee, R Bell, A Bularga, C Berry, P Chowienczyk, J P Greenwood, A D Hughes, K Khunti, L Kingham, C Lawson, K Mangion, N L Mills, A J Moss, S Neubauer, B Raman, A N Sattar, C L Sudlow, M Toshner,

Immunology

P J M Openshaw (Lead), D Altmann, J K Baillie, R Batterham, H Baxendale, N Bishop, C E Brightling, P C Calder, R A Evans, J L Heeney, T Hussell, P Klenerman, J M Lord, P Moss, S L Rowland-Jones, W Schwaeble, M G Semple, R S Thwaites, L Turtle, L V Wain, S Walmsley, D Wraith

Intensive Care

M J Rowland (Lead), A Rostron (Co-Lead), J K Baillie, B Connolly, A B Docherty, N I Lone, D F McAuley, D Parekh, A Rostron, J Simpson, C Summers

Lung Fibrosis

R G Jenkins (Co-Lead), J Porter (Co-Lead), R J Allen, R Aul, J K Baillie, S Barratt, P Beirne, J Blaikley, R C Chambers, N Chaudhuri, C Coleman, E Denneny, L Fabbri, P M George, M Gibbons, F Gleeson, B Gooptu, B Guillen Guio, I Hall, N A Hanley, L P Ho, E Hufton, J Jacob, I Jarrold, G Jenkins, S Johnson, M G Jones, S Jones, F Khan, P Mehta, J Mitchell, P L Molyneaux, J E Pearl, K Piper Hanley, K Poinasamy, J Quint, D Parekh, P Rivera-Ortega, L C Saunders, M G Semple, J Simpson, D Smith, M Spears, L G Spencer, S Stanel, I Stewart, A A R Thompson, D Thickett, R Thwaites, L V Wain, S Walker, S Walsh, J M Wild, D G Wootton, L Wright

Metabolic

S Heller (Co-Lead), M J Davies (Co-Lead), H Atkins, S Bain, J Dennis, K Ismail, D Johnston, P Kar, K Khunti, C Langenberg, P McArdle, A McGovern, T Peto, J Petrie, E Robertson, N Sattar, K Shah, J Valabhji, B Young

Pulmonary and Systematic Vasculature

L S Howard (Co-Lead), Mark Toshner (Co-Lead), C Berry, P Chowienczyk, D Lasserson, A Lawrie, O C Leavy, J Mitchell, L Price, J Quint, J Rossdale, N Sattar, C Sudlow, A A R Thompson, J M Wild, M Wilkins

Rehabilitation, Sarcopenia and Fatigue

S J Singh (Co-Lead), W D-C Man (Co-Lead), J M Lord (Co-Lead), N J Greening (Co-Lead), T Chalder (Co-Lead), J T Scott (Co-Lead), N Armstrong, E Baldry, M Baldwin, N Basu, M Beadsworth, L Bishop, C E Bolton, A Briggs, M Buch, G Carson, J Cavanagh, H Chinoy, E Daynes, S Defres, R A Evans, P Greenhaff, S Greenwood, M Harvie, M Husain, S MacDonald, A McArdle, H J C McAuley, A McMahon, M McNarry, G Mills, C Nolan, K O’Donnell, D Parekh, Pimm, J Sargent, L Sigfrid, M Steiner, D Stensel, A L Tan, J Whitney, D Wilkinson, D Wilson, M Witham, D G Wootton, T Yates

Renal

D Thomas (Lead), N Brunskill (Co-Lead), S Francis (Co-Lead), S Greenwood (Co-Lead), C Laing (Co-Lead), K Bramham, P Chowdhury, A Frankel, L Lightstone, S McAdoo, K McCafferty, M Ostermann, N Selby, C Sharpe, M Willicombe

Local Clinical Centre PHOSP-COVID trial staff

(listed in alphabetical order)

Airedale NHS Foundation Trust

A Shaw (PI), L Armstrong, B Hairsine, H Henson, C Kurasz, L Shenton

Aneurin Bevan University Health Board

S Fairbairn (PI), A Dell, N Hawkings, J Haworth, M Hoare, A Lucey, V Lewis, G Mallison, H Nassa, C Pennington, A Price, C Price, A Storrie, G Willis, S Young

Barts Health NHS Trust & Queen Mary University of London

P Pfeffer (PI), K Chong-James, C David, W Y James, A Martineau, O Zongo

Barnsley Hospital NHS Foundation Trust

A Sanderson (PI)

Belfast Health and Social Care Trust & Queen's University Belfast

L G Heaney (PI), C Armour, V Brown, T Craig, S Drain, B King, N Magee, D McAulay, E Major, L McGarvey, J McGinness, R Stone

Betsi Cadwaladr University Health Board

A Haggar (PI), A Bolger, F Davies, J Lewis, A Lloyd, R Manley, E McIvor, D Menzies, K Roberts, W Saxon, D Southern, C Subbe, V Whitehead

Borders General Hospital, NHS Borders

H El-Taweel (PI), J Dawson, L Robinson

Bradford Teaching Hospitals NHS Foundation Trust

D Saralaya (PI), L Brear, K Regan, K Storton

Cambridge University Hospitals NHS Foundation Trust, NIHR Cambridge Clinical Research Facility & University of Cambridge

J Fuld (PI), A Bermperi, I Cruz, K Dempsey, A Elmer, H Jones, S Jose, S Marciniak, M Parkes, C Ribeiro, J Taylor, M Toshner, L Watson, J Worsley

Cardiff and Vale University Health Board

R Sabit (PI), L Broad, A Buttress, T Evans, M Haynes, L Jones, L Knibbs, A McQueen, C Oliver, K Paradowski, J Williams

Chesterfield Royal Hospital NHS Trust

E Harris (PI), C Sampson

Cwm Taf Morgannwg University Health Board

C Lynch (PI), E Davies, C Evenden, A Hancock, K Hancock, M Rees, L Roche, N Stroud, T Thomas-Woods

East Cheshire NHS Trust

M Babores (PI), J Bradley-Potts, M Holland, N Keenan, S Shashaa, H Wassall

East Kent Hospitals University NHS Foundation Trust

E Beranova (PI), H Weston (PI), T Cosier, L Austin, J Deery, T Hazelton, C Price, H Ramos, R Solly, S Turney

Gateshead NHS Trust

L Pearce (PI), W McCormack, S Pugmire, W Stoker, A Wilson

Guy’s and St Thomas’ NHS Foundation Trust

N Hart (PI), LA Aguilar Jimenez, G Arbane, S Betts, K Bisnauthsing, A Dewar, P Chowdhury, A Dewar, G Kaltsakas, H Kerslake, MM Magtoto, P Marino, LM Martinez, M Ostermann, J Rossdale, TS Solano, E Wynn

Hampshire Hospitals NHS Foundation Trust

N Williams (PI), W Storrar (PI), M Alvarez Corral, A Arias, E Bevan, D Griffin, J Martin, J Owen,

S Payne, A Prabhu, A Reed, C Wrey Brown

Harrogate and District NHD Foundation Trust

C Lawson (PI), T Burdett, J Featherstone, A Layton, C Mills, L Stephenson,

Hull University Teaching Hospitals NHS Trust & University of Hull

N Easom (PI), P Atkin, K Brindle, M G Crooks, K Drury, R Flockton, L Holdsworth, A Richards, D L Sykes, S Thackray-Nocera, C Wright

Hywel Dda University Health Board

K E Lewis (PI), A Mohamed (PI), G Ross (PI), S Coetzee, K Davies, R Hughes, R Loosley, L O’Brien, Z Omar, H McGuinness, E Perkins, J Phipps, A Taylor, H Tench, R Wolf-Roberts

Imperial College Healthcare NHS Trust & Imperial College London

L S Howard (PI), O Kon (PI), D C Thomas (PI), S Anifowose, L Burden, E Calvelo, B Card, C Carr, E R Chilvers, D Copeland, P Cullinan, P Daly, L Evison, T Fayzan, H Gordon, S Haq, R G Jenkins, C King, K March, M Mariveles, L McLeavey, N Mohamed, S Moriera, U Munawar, J Nunag, U Nwanguma, L Orriss- Dib, A Ross, M Roy, E Russell, K Samuel, J Schronce, N Simpson, L Tarusan, C Wood, N Yasmin

Kettering General Hospital NHS Trust

R Reddy (PI), A-M, Guerdette, M Hewitt, K Warwick, S White

King’s College Hospital NHS Foundation Trust & Kings College London

A M Shah (PI), C J Jolley (PI), O Adeyemi, R Adrego, H Assefa-Kebede, J Breeze, M Brown, S Byrne, T Chalder, P Dulawan, N Hart, A Hayday, A Hoare, A Knighton, M Malim, S Patale, I Peralta, N Powell, A Ramos, K Shevket, F Speranza, A Te

Leeds Teaching Hospitals & University of Leeds

P Beirne (PI), A Ashworth, J Clarke, C Coupland, M Dalton, E Wade, C Favager, J Greenwood, J Glossop, L Hall, T Hardy, A Humphries, J Murira, D Peckham, S Plein, J Rangeley, G Saalmink, A L Tan, B Whittam, N Window, J Woods,

Lewisham & Greenwich NHS Trust

G Coakley (PI)

Liverpool University Hospitals NHS Foundation Trust & University of Liverpool

D G Wootton (PI), L Turtle (PI), L Allerton, AM All, M Beadsworth, A Berridge, J Brown, S Cooper, A Cross, S Defres, S L Dobson, J Earley, N French, W Greenhalf, H E Hardwick, K Hainey, J Hawkes, V Highett, S Kaprowska, AL Key, L Lavelle-Langham, N Lewis-Burke, G Madzamba, F Malein, S Marsh, C Mears, L Melling, M J Noonan, L Poll, J Pratt, E Richardson, A Rowe, M G Semple, V Shaw, K A Tripp, L O Wajero, S A Williams-Howard, J Wyles,

London North West University Healthcare NHS Trust

S N Diwanji (PI), P Papineni (PI), S Gurram, S Quaid, G F Tiongson, E Watson

Manchester University NHS Foundation Trust & University of Manchester

B Al-Sheklly (PI), A Horsley (PI), C Avram, J Blaikley, M Buch, N Choudhury, D Faluyi, T Felton, T Gorsuch, N A Hanley, T Hussell, Z Kausar, N Odell, R Osbourne, K Piper Hanley, K Radhakrishnan, S Stockdale

Newcastle upon Tyne Hospitals NHS Foundation Trust & University of Newcastle

A De Soyza (PI), C Echevarria (PI), A Ayoub, J Brown, G Burns, G Davies, H Fisher, C Francis, A Greenhalgh, P Hogarth, J Hughes, K Jiwa, G Jones, G MacGowan, D Price, A Sayer, J Simpson, H Tedd, S Thomas, S West, M Witham, S Wright, A Young

NHS Dumfries and Galloway

M J McMahon (PI), P Neill

NHS Greater Glasgow and Clyde Health Board & University of Glasgow

D Anderson (PI), H Bayes (PI), C Berry (PI), D Grieve (PI), I B McInnes (PI), N Basu, A Brown, A Dougherty, K Fallon, L Gilmour, K Mangion, A Morrow, K Scott, R Sykes

NHS Highland

E K Sage (PI), F Barrett, A Donaldson

NHS Lanarkshire

M Patel (PI), D Bell, A Brown, M Brown, R Hamil, K Leitch, L Macliver, J Quigley, A Smith, B Welsh

NHS Lothian & University of Edinburgh

G Choudhury (PI), J K Baillie, S Clohisey, A Deans, A B Docherty, J Furniss, E M Harrison, S Kelly, N I Lone, A Sheikh

NHS Tayside & University of Dundee

J D Chalmers (PI), D Connell, A Elliott, C Deas, J George, S Mohammed, J Rowland, A R Solstice, D Sutherland, C J Tee

North Bristol NHS Trust & University of Bristol

N Maskell (PI), D Arnold, S Barrett, H Adamali, A Dipper, S Dunn, A Morley, L Morrison, L Stadon, S Waterson, H Welch

North Middlesex Hospital NHS Trust

B Jayaraman (PI), T Light

Nottingham University Hospitals NHS Trust & University of Nottingham

C E Bolton (PI), P Almeida, J Bonnington, M Chrystal, C Dupont, P Greenhaff, A Gupta, L Howard, W Jang, S Linford, L Matthews, R Needham, A Nikolaidis, S Prosper, K Shaw, A K Thomas

Oxford University Hospitals NHS Foundation Trust & University of Oxford

L P Ho (PI), N M Rahman (PI), M Ainsworth, A Alamoudi, A Bates, A Bloss, A Burns, P Carter, J Chen, F Conneh, T Dong, R I Evans, E Fraser, X Fu, J R Geddes, F Gleeson, P Harrison, M Havinden-Williams, P Jezzard, N Kanellakis, I Koychev, P Kurupati, X Li, H McShane, C Megson, K Motohashi, S Neubauer, D Nicoll, G Ogg, E Pacpaco, M Pavlides, Y Peng, N Petousi, N Rahman, B Raman, M J Rowland, K Saunders, M Sharpe, N Talbot, E Tunnicliffe

Royal Brompton and Harefield Clinical Group, Guy’s and St Thomas’ NHS Foundation Trust.

W D-C Man (PI), B Patel (PI), R E Barker, D Cristiano, N Dormand, M Gummadi, S Kon, K Liyanage, C M Nolan, S Patel, O Polgar, P Shah, S J Singh, J A Walsh

Royal Free London NHS Foundation Trust

J R Hurst (PI), H Jarvis (PI), S Mandal (PI), S Ahmad, S E Brill, L Lim, D Matila, O Olaosebikan, C Singh

Royal Papworth Hospital NHS Foundation Trust

M Toshner (PI), H Baxendale, L Garner, C Johnson, J Mackie, A Michael, J Pack, K Paques, H Parfrey, J Parmar

Salford Royal NHS Foundation Trust

N Diar Bakerly (PI), P Dark, D Evans, E Hardy, A Harvey, D Holgate, S Knight, N Mairs, N Majeed, L McMorrow, J Oxton, J Pendlebury, C Summersgill, R Ugwuoke, S Whittaker

Salisbury NHS Foundation Trust

W Matimba-Mupaya (PI), S Strong-Sheldrake

Sheffield Teaching NHS Foundation Trust & University of Sheffield

S L Rowland-Jones (PI), A A R Thompson (Co PI), J Bagshaw, M Begum, K Birchall, R Butcher, H Carborn, F Chan, K Chapman, Y Cheng, L Chetham, C Clark, Z Coburn, J Cole, M Dixon, A Fairman, J Finnigan, H Foot, D Foote, A Ford, R Gregory, K Harrington, L Haslam, L Hesselden, J Hockridge, A Holbourn, B Holroyd-Hind, L Holt, A Howell, E Hurditch, F Ilyas, C Jarman, A Lawrie, E Lee, J-H Lee, R Lenagh, A Lye, I Macharia, M Marshall, A Mbuyisa, J McNeill, S Megson, J Meiring, L Milner, S Misra, H Newell, T Newman, C Norman, L Nwafor, D Pattenadk, M Plowright, J Porter, P Ravencroft, C Roddis, J Rodger, P Saunders, J Sidebottom, J Smith, L Smith, N Steele, G Stephens, R Stimpson, B Thamu, N Tinker, K Turner, H Turton, P Wade, S Walker, J Watson, I Wilson, A Zawia

St George’s University Hospitals NHS Foundation Trust

R Aul (PI), M Ali, A Dunleavy (PI), D Forton, N Msimanga, M Mencias, T Samakomva, S Siddique, J Teixeira, V Tavoukjian

Sherwood Forest Hospitals NHS Foundation Trust

J Hutchinson (PI), L Allsop, K Bennett, P Buckley, M Flynn, M Gill, C Goodwin, M Greatorex, H Gregory, C Heeley, L Holloway, M Holmes, J Kirk, W Lovegrove, TA Sewell, S Shelton, D Sissons, K Slack, S Smith, D Sowter, S Turner, V Whitworth, I Wynter

Shropshire Community Health NHS Trust

L Warburton (PI), S Painter, J Tomlinson

Somerset NHS Foundation Trust

C Vickers (PI), T Wainwright, D Redwood, J Tilley, S Palmer

Swansea Bay University Health Board

G A Davies (PI), L Connor, A Cook, T Rees, F Thaivalappil, C Thomas

Tameside and Glossop Integrated Care NHS Foundation

A Butt (PI), M Coulding, H Jones, S Kilroy, J McCormick, J McIntosh, H Savill, V Turner, J Vere

The Great Western Hospital Foundation Trust

E Fraile (PI), J Ugoji

The Hillingdon Hospitals NHS Foundation Trust

S S Kon (PI), H Lota, G Landers, M Nasseri, S Portukhay

The Rotherham NHS Foundation Trust

A Hormis (PI), A Daniels, J Ingham, L Zeidan

United Lincolnshire Hospitals NHS Trust

M Chablani (PI), L Osborne

University College London Hospital & University College London

M Marks (PI), J S Brown (PI), N Ahwireng, B Bang, D Basire, R C Chambers, A Checkley, R Evans, M Heightman, T Hillman, J Hurst, J Jacob, S Janes, R Jastrub, M Lipman, S Logan, D Lomas, M Merida Morillas, H Plant, J C Porter, K Roy, E Wall

University Hospital Birmingham NHS Foundation Trust & University of Birmingham

D Parekh (PI), N Ahmad Haider, C Atkin, R Baggott, M Bates, A Botkai, A Casey, B Cooper, J Dasgin, K Draxlbauer, N Gautam, J Hazeldine, T Hiwot, S Holden, K Isaacs, T Jackson, S Johnson, V Kamwa, D Lewis,

J M Lord, S Madathil, C McGhee, K Mcgee, A Neal, A Newton Cox, J Nyaboko, D Parekh, Z Peterkin, H Qureshi, L Ratcliffe, E Sapey, J Short, T Soulsby, J Stockley, Z Suleiman, T Thompson, M Ventura, S Walder, C Welch, D Wilson, S Yasmin, K P Yip

University Hospitals of Derby and Burton

P Beckett (PI) C Dickens, U Nanda

University Hospitals of Leicester NHS Trust & University of Leicester

C E Brightling (CI), R A Evans (PI), M Aljaroof, N Armstrong, H Arnold, H Aung, M Bakali, M Bakau, M Baldwin, M Bingham, M Bourne, C Bourne, N Brunskill, P Cairns, L Carr, A Charalambou, C Christie, M J Davies, S Diver, S Edwards, C Edwardson, O Elneima, H Evans, J Finch, S Glover, N Goodman, B Gootpu, N J Greening, K Hadley, P Haldar, B Hargadon, V C Harris, L Houchen-Wolloff, W Ibrahim, L Ingram, K Khunti, A Lea, D Lee, G P McCann, H J C McAuley, P McCourt, T Mcnally, G Mills, A Moss, W Monteiro, M Pareek, S Parker, A Rowland, A Prickett, I N Qureshi, R Russell, M Sereno, A Shikotra, S Siddiqui, A Singapuri, S J Singh, J Skeemer, M Soares, E Stringer, T Thornton, M Tobin, L V Wain, T J C Ward, F Woodhead, T Yates, A Yousuf

University Hospital Southampton NHS Foundation Trust & University of Southampton

M G Jones (PI), C Childs, R Djukanovic, S Fletcher, M Harvey, E Marouzet, B Marshall, R Samuel, T Sass, T Wallis, H Wheeler

Whittington Health NHS

R Dharmagunawardena (PI), E Bright, P Crisp, M Stern

Wirral University Teaching Hospital

A Wight (PI), L Bailey, A Reddington

Wrightington Wigan and Leigh NHS Trust

A Ashish (PI), J Cooper, E Robinson

Yeovil District Hospital NHS Foundation Trust

A Broadley (PI)

York & Scarborough NHS Foundation Trust

K Howard (PI), L Barman, C Brookes, K Elliott. L Griffiths, Z Guy, D Ionita, H Redfearn, C Sarginson

A Turnbull

Health and Care Research Wales

Y Ellis

London School of Hygiene & Tropical Medicine (LSHTM)

M Marks, A Briggs

NIHR Office for Clinical Research Infrastructure

K Holmes

Patient Public Involvement Leads

Asthma UK and British Lung Foundation Partnership - K Poinasamy, S Walker

Royal Surrey NHS Foundation Trust

M Halling-Brown

South London and Maudsley NHS Foundation Trust & Kings College London

G Breen, M Hotopf

Swansea University & Swansea Welsh Network

K Lewis, N Williams
